# Supplementary material for: Balancing the uncertain and unpredictable nature of possible zoonotic disease transmission with the value placed on animals: Findings from a qualitative study in Guinea
Source: PLOS Glob Public Health. 2024 Mar 28;4(3):e0001174. doi: 10.1371/journal.pgph.0001174 (PMC10977678; doi:10.1371/journal.pgph.0001174)
Supplement: S1 Appendix — (DOCX) [file pgph.0001174.s001.docx]

**Guide pour les Groupes de Discussion**

**GENERAL POPULATION**

**Introduction (5 min)**

Merci pour votre présence aujourd’hui.

**1.** **Nous allons faire un tour et s'il vous plaît,** **présentez-vous en nous disant votre prénom et quelque chose que vous appréciez à propos de la vie dans cette communauté.**

**Perceptions des animaux (15 min)**

Aujourd'hui, nous allons discuter les animaux. Voici différents animaux avec lesquels les gens les gens interagissent au cours d'une journée typique. *[Répartir au hasard les participants en 2-3 groupes de 2 à 4 personnes.]*

**2. En votre petite groupe, discuter et arrengez les cartes en 2 ou 3 piles. Pour chaque pile, choisir un nom bref pour le groupe qui décrit ce qui les unissent.**

*Bovins, moutons, chèvres, chauves-souris, poulets, porcs, rongeurs, chats, chiens*

*[Donnez 3-4 minutes pour le triage.]*

**Maintenant, chaque groupe peut nous montrer ses groupes et nous dire le nom des groupes.** *[10 minutes total. Donnez à chaque petite groupe 1-2 minutes pour présenter son travail à la plénière.]*

*Sondes possibles :*

- Qu'est-ce que ces animaux [pointer vers un groupe] ont en commun ?
- En quoi sont-ils différents de ces animaux ?

**Connaissance et perception de risque (5-10 min)**

Aujourd'hui, nous allons parler de la relation entre les animaux et les gens de votre communauté.

**3. Vous avez déjà parlé quelques** **maladies causées par les animaux que vous avez entendu parler. Qu’est-ce que vous savez de ces maladies ?**

*Sondes possibles :*

- Que savez-vous de la façon dont ces maladies se transmettent des animaux aux humains ?
- Que savez-vous de la rage ? *[Demandez seulement si personne n'a pas déjà parlé de la rage.]*

**4. Dans quelle mesure les gens sont-ils préoccupés par les maladies qui proviennent des animaux ?**

*Sondes possibles :*

- Comment cela se compare-t-il à d'autres soucis dans leur vie ?
- Qu'est-ce qui pourrait rendre une personne de cette communauté préoccupée par de telles maladies qui passent d'animaux à l'homme ?

**Comportements de prévention (60-65 min)**

**5. Qu'est-ce qu'on vous a dit que les gens dans votre communauté font pour empêcher les maladies de passer des animaux aux humains ?**

*Sondes possibles :*

- Autres moyens ?
- Qu'en est-il des croyances locales sur la façon de se protéger contre les maladies qui viennent des animaux ?

**6. Maintenant, on va faire une dynamique et organiser ces cartes en groupe. Chaque carte contient l'image d'une action. A chaque action, je leur demanderai si c'est faisable. Ce qui signifie que si les gens trouvent cela facile, ils peuvent le faire, si le matériel nécessaire est facile à obtenir, etc. Ensuite, nous discuterons de la question de savoir si l'action servira à prévenir les maladies causées par les animaux.**

[*Examinez et discutez chaque pratique en particulier. Lire la description de la première image*]

| **A** | Gardez les animaux séparés des lieux d’habitation |
| --- | --- |
| **C** | Évitez les morsures de chien |
| **E** | Vacciner les animaux (comme chiens, poulets, vaches …) |
| **F** | Faire bouillir le lait pendant 30 min avant de boire |
| **G** | Stériliser les couteaux et les surfaces utilisées pour couper la viande fraiche |
| **H** | Bien cuire la viande et seulement manger la viande bien cuite |
| **I** | Éviter de manger de la viande d’animaux malades |
| **J** | Éviter de manger de la viande de brousse |
| **K** | Couvrir les coupures ou les blessures sur la peau lorsque vous manipulez des animaux |
| **O** | Rangez les aliments dans des contenants couverts pour les protéger des rongeurs |

*[Après avoir lu la description de la première image, discutez avec le groupe]* **Que pensez-vous si les gens dans votre communauté pensent que le comportement est facile à réaliser ?**

*Sondes possibles :*

- Par exemple, les gens trouvent-ils cela facile [action X] ? Est-ce que les gens peuvent le faire ? Normalement, dans la vraie vie, est-ce que les gens le font ? Toutes les choses nécessaires sont-elles accessibles ?

*[Après avoir lu la description de la première image, discutez avec le groupe]*

**Que pensez-vous si le comportement est facile à réaliser pour les gens dans votre communauté C'est-à-dire que, dans le monde actuel, est-ce-que les gens dans votre communauté le font ?**

*[Vous pouvez donner des exemples de différents aspects de faisabilité qui s'appliquent à l'action en question pour clarifier un peu plus, comme certains des aspects suivants]*

- - Nombre d'étapes nécessaires à la réalisation de l'action
  - Comme il est facile ou difficile de faire les étapes
  - Comme il est facile ou difficile d'organiser ou de planifier l'action (p. ex. prendre des rendez-vous).
  - Le temps nécessaire pour faire l'action (c'est quelque chose que vous n'avez à faire qu'une seule fois ou que vous devez faire et refaire, le temps passé à faire l'action que vous ne faites rien d'autre, etc.)
  - Comme il est facile ou difficile d'obtenir ce dont vous avez besoin pour faire l'action
  - Coût/prix des matériaux (p.ex. savon)
  - La mesure dans laquelle l'action est une norme dans la communauté
  - Ce que la culture dicte à propos de l'action

*Sondes possibles :*

- Autres opinions ?
- Par exemple, les gens trouvent-ils cela facile [action X] ?
- Est-ce que les gens dans votre communauté réalisent de le faire ?

*[Écouter l'opinion de certains membres du groupe sur la faisabilité d'une action. En raison du temps, tout le monde n'a pas à donner son opinion. Calculer 3 minutes par action.]*

**Maintenant, levez la main si vous pensez que** *[l’action]***...**

- **est très facile de réaliser**
- **est plus ou moins facile à réaliser**
- **n'est pas facile à réaliser**

*[Ensuite, placez l'action sur le tableau à feuilles et prenez la deuxième image de la même action pour commencer les questions sur l'efficacité. Écoutez l'opinion de certains membres du groupe sur l'efficacité de l'action X. A cause du temps, tout le monde n'a pas besoin de donner son opinion.]*

**Supposons maintenant que** *[nommez quelques défis qu'ils ont mentionnés pour la faisabilité]* **ne soient plus un problème et qu'il soit facile à réaliser le comportement. Que pensez-vous si [l'action X] est utile pour la prévention des zoonoses ?**

*Sondes possibles :*

- Autres opinions ?
- Comment cela fonctionne-t-il ?
- Qu'est-ce qui vous fait croire qu'il est efficace/qu'il fonctionne bien pour prévenir la maladie ?

*[Calculer 3 minutes par action.]*

**Maintenant, levez la main si vous pensez que** *[l’action]* **pour la prévention des maladies zoonoses. Levez la main si vous pensez...**

- **est très utile pour la prévention des maladies zoonoses**
- **est plus ou moins utile pour la prévention des maladies zoonoses**
- **N’est pas utile pour la prévention des maladies zoonoses**

*[Ensuite, placez l'action sur le tableau à feuilles mobiles. Répétez l'opération avec la carte suivante. Après les premières cartes, lorsque le groupe est plus à l'aise avec l'activité, il n'est pas nécessaire d'expliquer chaque groupe de « un peu, plus ou moins et beaucoup ». Vous pouvez dire au groupe : « Maintenant, il faut choisir. Ceux qui pensent un peu, plus ou moins ou beaucoup.»]*

**Hygiène (5 min)**

Maintenant, je voudrais parler de la propreté.

**7. Quand s'agit-il de traiter avec des animaux, quand est-il important de se laver soi-même ?**

*Sondes possibles :*

- Après quelles interactions avec les animaux devez-vous vous laver ?
- Quelles parties de votre corps est-il important de nettoyer après avoir soigné des animaux ou de la viande ?
- Quand vous considérez-vous assez propre ? *[Par exemple, n'a pas l'air sale, odeur, etc.]*

**8. Pensez à l'endroit où vous manipulez de la viande crue : Quand est-il important de laver cette zone ?**

*Sondes possibles :*

- Comment quelqu'un devrait le laver ? *[Produits, processus]*
- Comment décideriez-vous si quelque chose mérite d'être nettoyé ?
- Comment décideriez-vous si c'est assez propre ?

**Chercher de soins (10 min)**

Maintenant, je voudrais parler des cas où les animaux sont malades.

**8. Quels sont les signes ou les symptômes qui pourraient permettre aux gens dans votre communauté de suspecter chez un animal une maladie ?**

**10. Qu’est-ce que font les gens dans votre communauté lorsqu’ils suspectent une maladie chez un de leurs animaux ?**

*Sondes possibles :*

- Pourquoi font-ils cela ?
- Que pourraient-ils faire d’autre ? Pourquoi ?

**11. Qu'est-ce qui motive les gens à appeler un vétérinaire ?**

*Sondes possibles :*

- Dans quelles situations ?
- Pourquoi quelqu'un préférerait-il ne pas appeler un vétérinaire ?

**12. Qu'est-ce qui motive les gens à faire des tests sur les animaux pour voir s’ils sont malades ?**

*Sondes possibles :*

- Pour quelles maladies ? Dans quelles situations ?
- Quels types de personnes sont plus susceptibles de font faire des tests sur les animaux pour voir s’ils sont malades ?
- Pourquoi quelqu'un préférerait-il ne pas faire des tests ?

**13. Dans quels types de situations une personne peut-elle tuer les animaux malades ?**

*Sondes possibles :*

- Qu'est-ce qui peut rendre difficile pour une personne de tuer les animaux malades ?
- Que font-ils avec les animaux lorsqu’ils les ont tués ?
- Autres situations ?

On a parlé d'animaux malades. Maintenant, j'aimerais parler du moment où les humains tombent malades.

**14. Quels sont les facteurs qui influencent la recherche de soins de santé pour les humains ?**

*Sondes possibles*

- Quels symptômes peuvent inciter quelqu'un à se rendre dans un centre de santé pour un membre de sa famille ?
- Autres facteurs ?

**Sources de communication (10 min)**

Maintenant, j’aimerais maintenant parler un peu de la façon dont les gens de cette communauté obtiennent de l'information sur la santé.

**15. Laquelle de ces sources est la meilleure source d’information sur votre santé et de celle de votre famille ?**

*Sondes possibles :*

- Qu'est-ce qui change quand il s'agit de la santé de vos animaux ?
- Comment les choses changent-elles lorsqu'il s'agit d'une situation de crise ?

**16. Supposons que vous voyez ou entendez quelques informations sur la santé. Comment décidez-vous si ces informations sont fiables ?**

*Sondes possibles :*

- C'est-à-dire, quelles sont les caractéristiques de l'information qui vous aident à décider si l'information est fiable ?
- Sonder les canaux *[par exemple, radio, télévision, internet, téléphone, réunions communautaires]*, les sources d'information *[leaders communautaires, leaders religieux, voisins, prestataires de soins de santé, guérisseurs traditionnels]*, le contenu de l'information elle-même
- **Comment les choses changent-elles en temps de crise ?**

**17. Selon vous, quels sont les meilleurs moyens pour les autorités sanitaires de communiquer rapidement l'information à votre communauté en cas de crise ?**

*Sondes possibles :*

- Pendant une crise, quel type d'information serait important pour vous ?
- Quels autres moyens les autorités sanitaires pourraient-ils prendre pour communiquer rapidement ce genre d'information à votre communauté ?

**Conclusion**

Merci de m'avoir parlé aujourd'hui. Vous avez partagé *[Résumez ce dont vous avez discuté avec les participants].*

**18. Y a-t-il autre chose que vous aimeriez nous faire savoir sur votre communauté et comment les gens interagissent avec les animaux ?**

Merci beaucoup pour le temps que vous nous avez consacré.

**Guide pour les Groupes de Discussion**

**BOUCHERS**

**Introduction (5 min)**

Merci pour votre présence aujourd’hui.

**1.** **Nous allons faire un tour et s'il vous plaît,** **présentez-vous en nous disant votre prénom et quelque chose que vous appréciez à propos de la vie dans cette communauté.**

**Perceptions des animaux (15 min)**

Aujourd'hui, nous allons discuter les animaux. Voici différents animaux avec lesquels les gens les gens interagissent au cours d'une journée typique. *[Répartir au hasard les participants en 2-3 groupes de 2 à 4 personnes.]*

**2. En votre petite groupe, discuter et arrengez les cartes en 2 ou 3 piles. Pour chaque pile, choisir un nom bref pour le groupe qui décrit ce qui les unissent.**

*Bovins, moutons, chèvres, chauves-souris, poulets, porcs, rongeurs, chats, chiens*

*[Donnez 3-4 minutes pour le triage.]*

**Maintenant, chaque groupe peut nous montrer ses groupes et nous dire le nom des groupes.** *[10 minutes total. Donnez à chaque petite groupe 1-2 minutes pour présenter son travail à la plénière.]*

*Sondes possibles :*

- Qu'est-ce que ces animaux *[pointer vers un groupe]* ont en commun ?
- En quoi sont-ils différents de ces animaux ?

**Connaissance et perception de risque (5-10 min)**

Aujourd'hui, nous allons parler de la relation entre les animaux et les bouchers comme vous.

**3. Vous avez déjà parlé quelques** **maladies causées par les animaux que vous avez entendu parler. Qu’est-ce que vous savez de ces maladies ?**

*Sondes possibles :*

- Que savez-vous de la façon dont ces maladies se transmettent des animaux aux humains ?
- Que savez-vous de la rage ? *[Demandez seulement si personne n'a pas déjà parlé de la rage.]*

**4. Dans quelle mesure les bouchers comme vous sont-ils préoccupés par les maladies qui proviennent des animaux ?**

*Sondes possibles :*

- Comment cela se compare-t-il à d'autres soucis dans votre vie ?
- Qu'est-ce qui pourrait rendre un boucher comme vous préoccupée par de telles maladies qui passent d'animaux à l'homme ?

**Comportements de prévention (60-65 min)**

**5. Qu'est-ce qu'on vous a dit que les bouchers comme vous font pour empêcher les maladies de passer des animaux aux humains ?**

*Sondes possibles :*

- Autres moyens ?
- Qu'en est-il des croyances locales sur la façon de se protéger contre les maladies qui viennent des animaux ?

**6. Maintenant, on va faire une dynamique et organiser ces cartes en groupe. Chaque carte contient l'image d'une action. A chaque action, je leur demanderai si c'est faisable pour les bouchers comme vous. Ce qui signifie que s’ils trouvent cela facile, ils peuvent le faire, si le matériel nécessaire est facile à obtenir, etc. Ensuite, nous discuterons de la question de savoir si l'action servira à prévenir les maladies causées par les animaux.**

[*Examinez et discutez chaque pratique en particulier. Lire la description de la première image*]

| **A** | Gardez les animaux séparés des lieux d’habitation |
| --- | --- |
| **B** | Désinfecter les enclos pour animaux |
| **C** | Évitez les morsures de chien |
| **G** | Stériliser les couteaux et les surfaces utilisées pour couper la viande fraiche |
| **I** | Éviter de manger de la viande d’animaux malades |
| **J** | Éviter de manger de la viande de brousse |
| **K** | Couvrir les coupures ou les blessures sur la peau lorsque vous manipulez des animaux |
| **L** | Porter une tenue de protection avant de toucher les carcasses |
| **M** | Enterrer les carcasses d’animaux malades et les fœtus avortés |

*[Après avoir lu la description de la première image, discutez avec le groupe]* **Que pensez-vous si le comportement est facile à réaliser pour les bouchers comme vous ? C'est-à-dire que, dans le monde actuel, est-ce-que les bouchers le font ?**

*[Vous pouvez donner des exemples de différents aspects de faisabilité qui s'appliquent à l'action en question pour clarifier un peu plus, comme certains des aspects suivants]*

- - Nombre d'étapes nécessaires à la réalisation de l'action
  - Comme il est facile ou difficile de faire les étapes
  - Comme il est facile ou difficile d'organiser ou de planifier l'action (p. ex. prendre des rendez-vous).
  - Le temps nécessaire pour faire l'action (c'est quelque chose que vous n'avez à faire qu'une seule fois ou que vous devez faire et refaire, le temps passé à faire l'action que vous ne faites rien d'autre, etc.)
  - Comme il est facile ou difficile d'obtenir ce dont vous avez besoin pour faire l'action
  - Coût/prix des matériaux (p.ex. savon)
  - La mesure dans laquelle l'action est une norme avec les bouchers
  - Ce que la culture dicte à propos de l'action

*Sondes possibles :*

- Autres opinions ?
- Par exemple, les bouchers trouvent-ils cela facile [action X] ?
- Est-ce que les bouchers réalisent de le faire ?

*[Écouter l'opinion de certains membres du groupe sur la faisabilité d'une action. En raison du temps, tout le monde n'a pas à donner son opinion. Calculer 3 minutes par action.]*

**Maintenant, levez la main si vous pensez que, pour les bouchers comme vous,** *[l’action]***...**

- **est très facile de réaliser**
- **est plus ou moins facile à réaliser**
- **n'est pas facile à réaliser**

*[Ensuite, placez l'action sur le tableau à feuilles et prenez la deuxième image de la même action pour commencer les questions sur l'efficacité. Écoutez l'opinion de certains membres du groupe sur l'efficacité de l'action X. A cause du temps, tout le monde n'a pas besoin de donner son opinion.]*

**Supposons maintenant que** *[nommez quelques défis qu'ils ont mentionnés pour la faisabilité]* **ne soient plus un problème et qu'il soit facile à réaliser le comportement. Que pensez-vous si [l'action X] est utile pour la prévention des zoonoses ?**

*Sondes possibles :*

- Autres opinions ?
- Comment cela fonctionne-t-il ?
- Qu'est-ce qui vous fait croire qu'il est efficace/qu'il fonctionne bien pour prévenir la maladie ?

*[Calculer 3 minutes par action.]*

**Maintenant, levez la main si vous pensez que** *[l’action]* **pour la prévention des maladies zoonoses. Levez la main si vous pensez...**

- **est très utile pour la prévention des maladies zoonoses**
- **est plus ou moins utile pour la prévention des maladies zoonoses**
- **N’est pas utile pour la prévention des maladies zoonoses**

*[Ensuite, placez l'action sur le tableau à feuilles mobiles. Répétez l'opération avec la carte suivante. Après les premières cartes, lorsque le groupe est plus à l'aise avec l'activité, il n'est pas nécessaire d'expliquer chaque groupe de « un peu, plus ou moins et beaucoup ». Vous pouvez dire au groupe : « Maintenant, il faut choisir. Ceux qui pensent un peu, plus ou moins ou beaucoup.»]*

**Hygiène (5 min)**

Maintenant, je voudrais parler de la propreté.

**7. Quand s'agit-il de traiter avec des animaux, quand est-il important de se laver soi-même ?**

*Sondes possibles :*

- Après quelles interactions avec les animaux devez-vous vous laver ?
- Quelles parties de votre corps est-il important de nettoyer après avoir soigné des animaux ou de la viande ?
- Quand vous considérez-vous assez propre ? *[Par exemple, n'a pas l'air sale, odeur, etc.]*

**8. Pensez à l'endroit où vous manipulez de la viande crue : Quand est-il important de laver cette zone ?**

*Sondes possibles :*

- Comment quelqu'un devrait le laver ? *[Produits, processus]*
- Comment décideriez-vous si quelque chose mérite d'être nettoyé ?
- Comment décideriez-vous si c'est assez propre ?

**Chercher de soins (10 min)**

Maintenant, je voudrais parler des cas où les animaux sont malades.

**9. Quels sont les signes ou les symptômes qui pourraient permettre aux bouchers comme vous de suspecter chez un animal une maladie ?**

**10. Qu’est-ce que font les bouchers comme vous lorsqu’ils suspectent une maladie chez un de leurs animaux ?**

*Sondes possibles :*

- Pourquoi font-ils cela ?
- Que pourraient-ils faire d’autre ? Pourquoi ?

**11. Qu'est-ce qui motive les bouchers comme vous à appeler un vétérinaire ?**

*Sondes possibles :*

- Dans quelles situations ?
- Pourquoi quelqu'un préférerait-il ne pas appeler un vétérinaire ?
- Lorsqu’un de vos animaux est malade, quel sont les produits que vous leur administrez pour les soigner ? Comment choisissez – vous ces produits ?

**12. Qu'est-ce qui motive les bouchers comme vous à faire des tests sur les animaux pour voir s’ils sont malades ?**

*Sondes possibles :*

- Pour quelles maladies ? Dans quelles situations ?
- Pourquoi quelqu'un préférerait-il ne pas faire des tests ?

**13. Dans quels types de situations un boucher peut-il tuer les animaux malades ?**

*Sondes possibles :*

- Qu'est-ce qui peut rendre difficile pour une personne de tuer les animaux malades ?
- Que font-ils avec les animaux lorsqu’ils les ont tués ?
- Autres situations ?

On a parlé d'animaux malades. Maintenant, j'aimerais parler du moment où les humains tombent malades.

**14. Quels sont les facteurs qui influencent la recherche de soins pour les humains pour les bouchers comme vous ?**

*Sondes possibles :*

- Quels symptômes peuvent inciter quelqu'un à se rendre dans un centre de santé pour un membre de sa famille ?
- Autres facteurs ?

**Sources de communication (10 min)**

Maintenant, j’aimerais maintenant parler un peu de la façon dont les gens de cette communauté obtiennent de l'information sur la santé.

**15. Laquelle de ces sources est la meilleure source d’information sur votre santé et de celle de votre famille ?**

*Sondes possibles :*

- Qu'est-ce qui change quand il s'agit de la santé de vos animaux ?
- **Comment les choses changent-elles en temps de crise ?**

**16. Supposons que vous voyez ou entendez quelques informations sur la santé. Comment décidez-vous si ces informations sont fiables ?**

*Sondes possibles :*

- C'est-à-dire, quelles sont les caractéristiques de l'information qui vous aident à décider si l'information est fiable ?
- Sonder les canaux *[par exemple, radio, télévision, internet, téléphone, réunions communautaires]*, les sources d'information *[leaders communautaires, leaders religieux, voisins, prestataires de soins de santé, guérisseurs traditionnels]*, le contenu de l'information elle-même
- **Comment les choses changent-elles lorsqu'il s'agit d'une situation de crise ?**

**17. Selon vous, quels sont les meilleurs moyens pour les autorités sanitaires de communiquer rapidement l'information à les bouchers comme vous en cas de crise ?**

*Sondes possibles :*

- Pendant une crise, quel type d'information serait important pour vous ?
- Quels autres moyens les autorités sanitaires pourraient-ils prendre pour communiquer rapidement ce genre d'information aux bouchers comme vous ?

**Conclusion (5 min)**

Merci de m'avoir parlé aujourd'hui. Vous avez partagé *[Résumez ce dont vous avez discuté avec les participants].*

**18. Y a-t-il autre chose que vous aimeriez nous faire savoir sur les bouchers comme vous et comment ils interagissent avec les animaux ?**

Merci beaucoup pour le temps que vous nous avez consacré.

**Guide pour les Groupes de Discussion**

**CHASSEURS**

**Introduction (5 min)**

Merci pour votre présence aujourd’hui.

**1.** **Nous allons faire un tour et s'il vous plaît,** **présentez-vous en nous disant votre prénom et quelque chose que vous appréciez à propos de la vie dans cette communauté.**

**Perceptions des animaux (15 min)**

Aujourd'hui, nous allons discuter les animaux. Voici différents animaux avec lesquels les gens les gens interagissent au cours d'une journée typique. *[Répartir au hasard les participants en 2-3 groupes de 2 à 4 personnes.]*

**2. En votre petite groupe, discuter et arrengez les cartes en 2 ou 3 piles. Pour chaque pile, choisir un nom bref pour le groupe qui décrit ce qui les unissent.**

*Bovins, moutons, chèvres, chauves-souris, poulets, porcs, rongeurs, chats, chiens*

*[Donnez 3-4 minutes pour le triage.]*

**Maintenant, chaque groupe peut nous montrer ses groupes et nous dire le nom des groupes.** *[10 minutes total. Donnez à chaque petite groupe 1-2 minutes pour présenter son travail à la plénière.]*

*Sondes possibles :*

- Qu'est-ce que ces animaux *[pointer vers un groupe]* ont en commun ?
- En quoi sont-ils différents de ces animaux ?

**Connaissance et perception de risque (5-10 min)**

Aujourd'hui, nous allons parler de la relation entre les animaux et les chasseurs comme vous.

**3. Vous avez déjà parlé quelques** **maladies causées par les animaux que vous avez entendu parler. Qu’est-ce que vous savez de ces maladies ?**

*Sondes possibles :*

- Que savez-vous de la façon dont ces maladies se transmettent des animaux aux humains ?
- Que savez-vous de la rage ? *[Demandez seulement si personne n'a pas déjà parlé de la rage.]*

**4. Dans quelle mesure les chasseurs comme vous sont-ils préoccupés par les maladies qui proviennent des animaux ?**

*Sondes possibles :*

- Comment cela se compare-t-il à d'autres soucis dans leur vie ?
- Qu'est-ce qui pourrait rendre un chasseur comme vous préoccupée par de telles maladies qui passent d'animaux à l'homme ?

**Comportements de prévention (60-65 min)**

**5. Qu'est-ce qu'on vous a dit que les chasseurs comme vous font pour empêcher les maladies de passer des animaux aux humains ?**

*Sondes possibles :*

- Autres moyens ?
- Qu'en est-il des croyances locales sur la façon de se protéger contre les maladies qui viennent des animaux ?

**6. Maintenant, on va faire une dynamique et organiser ces cartes en groupe. Chaque carte contient l'image d'une action. A chaque action, je leur demanderai si c'est faisable pour les chasseurs comme vous. Ce qui signifie que s’ils trouvent cela facile, ils peuvent le faire, si le matériel nécessaire est facile à obtenir, etc. Ensuite, nous discuterons de la question de savoir si l'action servira à prévenir les maladies causées par les animaux.**

[*Examinez et discutez chaque pratique en particulier. Lire la description de la première image*]

| **A** | Gardez les animaux séparés des lieux d’habitation |
| --- | --- |
| **B** | Désinfecter les enclos pour animaux |
| **C** | Évitez les morsures de chien |
| **G** | Stériliser les couteaux et les surfaces utilisées pour couper la viande fraiche |
| **I** | Éviter de manger de la viande d’animaux malades |
| **J** | Éviter de manger de la viande de brousse |
| **K** | Couvrir les coupures ou les blessures sur la peau lorsque vous manipulez des animaux |
| **L** | Porter une tenue de protection avant de toucher les carcasses |
| **M** | Enterrer les carcasses d’animaux malades et les fœtus avortés |
| **N** | Éviter de manger des fruits déjà en partie consommés par un animal |

*[Après avoir lu la description de la première image, discutez avec le groupe]*

**Que pensez-vous si le comportement est facile à réaliser pour les bouchers comme vous ? C'est-à-dire que, dans le monde actuel, est-ce-que les bouchers le font ?**

*[Vous pouvez donner des exemples de différents aspects de faisabilité qui s'appliquent à l'action en question pour clarifier un peu plus, comme certains des aspects suivants]*

- - Nombre d'étapes nécessaires à la réalisation de l'action
  - Comme il est facile ou difficile de faire les étapes
  - Comme il est facile ou difficile d'organiser ou de planifier l'action (p. ex. prendre des rendez-vous).
  - Le temps nécessaire pour faire l'action (c'est quelque chose que vous n'avez à faire qu'une seule fois ou que vous devez faire et refaire, le temps passé à faire l'action que vous ne faites rien d'autre, etc.)
  - Comme il est facile ou difficile d'obtenir ce dont vous avez besoin pour faire l'action
  - Coût/prix des matériaux (p.ex. savon)
  - La mesure dans laquelle l'action est une norme avec les bouchers
  - Ce que la culture dicte à propos de l'action

*Sondes possibles :*

- Autres opinions ?
- Par exemple, les bouchers trouvent-ils cela facile [action X] ?
- Est-ce que les bouchers réalisent de le faire ?

*[Écouter l'opinion de certains membres du groupe sur la faisabilité d'une action. En raison du temps, tout le monde n'a pas à donner son opinion. Calculer 3 minutes par action.]*

**Maintenant, levez la main si vous pensez que, pour les chasseurs comme vous,** *[l’action]***...**

- **est très facile de réaliser**
- **est plus ou moins facile à réaliser**
- **n'est pas facile à réaliser**

*[Ensuite, placez l'action sur le tableau à feuilles et prenez la deuxième image de la même action pour commencer les questions sur l'efficacité. Écoutez l'opinion de certains membres du groupe sur l'efficacité de l'action X. A cause du temps, tout le monde n'a pas besoin de donner son opinion.]*

**Supposons maintenant que** *[nommez quelques défis qu'ils ont mentionnés pour la faisabilité]* **ne soient plus un problème et qu'il soit facile à réaliser le comportement. Que pensez-vous si [l'action X] est utile pour la prévention des zoonoses ?**

*Sondes possibles :*

- Autres opinions ? Comment cela fonctionne-t-il ?
- Qu'est-ce qui vous fait croire qu'il est efficace/qu'il fonctionne bien pour prévenir la maladie ?

*[Calculer 3 minutes par action.]*

**Maintenant, levez la main si vous pensez que** *[l’action]* **pour la prévention des maladies zoonoses. Levez la main si vous pensez...**

- **est très utile pour la prévention des maladies zoonoses**
- **est plus ou moins utile pour la prévention des maladies zoonoses**
- **N’est pas utile pour la prévention des maladies zoonoses**

*[Ensuite, placez l'action sur le tableau à feuilles mobiles. Répétez l'opération avec la carte suivante. Après les premières cartes, lorsque le groupe est plus à l'aise avec l'activité, il n'est pas nécessaire d'expliquer chaque groupe de « un peu, plus ou moins et beaucoup ». Vous pouvez dire au groupe : « Maintenant, il faut choisir. Ceux qui pensent un peu, plus ou moins ou beaucoup.»]*

**Hygiène (5 min)**

Maintenant, je voudrais parler de la propreté.

**7. Quand s'agit-il de traiter avec des animaux, quand est-il important de se laver soi-même ?**

*Sondes possibles :*

- Après quelles interactions avec les animaux devez-vous vous laver ?
- Quelles parties de votre corps est-il important de nettoyer après avoir soigné des animaux ou de la viande ?
- Quand vous considérez-vous assez propre ? *[Par exemple, n'a pas l'air sale, etc.]*

**8. Pensez à l'endroit où vous manipulez de la viande crue : Quand est-il important de laver cette zone ?**

*Sondes possibles :*

- Comment quelqu'un devrait le laver ? *[Produits, processus]*
- Comment décideriez-vous si quelque chose mérite d'être nettoyé ?
- Comment décideriez-vous si c'est assez propre ?

**Chercher de soins (10 min)**

Maintenant, je voudrais parler des cas où les animaux sont malades.

**9. Quels sont les signes ou les symptômes qui pourraient permettre aux chasseurs comme vous de suspecter chez un animal une maladie ?**

**10. Qu’est-ce que font les chasseurs comme vous lorsqu’ils suspectent une maladie chez un animal ?**

*Sondes possibles :*

- Pourquoi font-ils cela ?
- Que pourraient-ils faire d’autre ? Pourquoi ?

**11. Quels sont les facteurs qui influencent le recours aux soins de santé pour les chasseurs comme vous ?**

*Sondes possibles :*

- Quels symptômes peuvent inciter quelqu'un à se rendre dans un centre de santé pour un membre de sa famille ? Autres facteurs ?

**Sources de communication (10 min)**

Maintenant, j’aimerais maintenant parler un peu de la façon dont les gens de cette communauté obtiennent de l'information sur la santé.

**12. Laquelle de ces sources est la meilleure source d’information sur votre santé et de celle de votre famille ?**

*Sondes possibles :*

- Qu'est-ce qui change quand il s'agit de la santé de vos animaux ?
- **Comment les choses changent-elles en temps de crise ?**

**13. Supposons que vous voyez ou entendez quelques informations sur la santé. Comment décidez-vous si ces informations sont fiables ?**

*Sondes possibles :*

- C'est-à-dire, quelles sont les caractéristiques de l'information qui vous aident à décider si l'information est fiable ?
- Sonder les canaux *[par exemple, radio, télévision, internet, téléphone, réunions communautaires]*, les sources d'information *[leaders communautaires, leaders religieux, voisins, prestataires de soins de santé, guérisseurs traditionnels]*, le contenu de l'information elle-même
- **Comment les choses changent-elles en temps de crise ?**

**14. Selon vous, quels sont les meilleurs moyens pour les autorités sanitaires de communiquer rapidement l'information aux chasseurs comme vous en cas de crise ?**

*Sondes possibles :*

- Pendant une crise, quel type d'information serait important pour vous ?
- Quels autres moyens les autorités sanitaires pourraient-ils prendre pour communiquer rapidement ce genre d'information aux chasseurs comme vous ?

**Conclusion (5 min)**

Merci de m'avoir parlé aujourd'hui. Vous avez partagé *[Résumez ce dont vous avez discuté avec les participants].*

**15. Y a-t-il autre chose que vous aimeriez nous faire savoir sur les chasseurs comme vous et comment ils interagissent avec les animaux ?**

Merci beaucoup pour le temps que vous nous avez consacré.

**Guide pour les Groupes de Discussion**

**ELEVEURS & VENDEURS DES ANIMAUX**

**Introduction (5 min)**

Merci pour votre présence aujourd’hui.

**1.** **Nous allons faire un tour et s'il vous plaît,** **présentez-vous en nous disant votre prénom et quelque chose que vous appréciez à propos de la vie dans cette communauté.**

**Perceptions des animaux (15 min)**

Aujourd'hui, nous allons discuter les animaux. Voici différents animaux avec lesquels les gens les gens interagissent au cours d'une journée typique. *[Répartir au hasard les participants en 2-4 groupes de 2 à 4 personnes.]*

**2. En votre petite groupe, discuter et arrengez les cartes en 2 ou 3 piles. Pour chaque pile, choisir un nom bref pour le groupe qui décrit ce qui les unissent.**

*Bovins, moutons, chèvres, chauves-souris, poulets, porcs, rongeurs, chats, chiens*

*[Donnez 3-4 minutes pour le triage.]*

**Maintenant, chaque groupe peut nous montrer ses groupes et nous dire le nom des groupes.** *[10 minutes total. Donnez à chaque petite groupe 1-2 minutes pour présenter son travail à la plénière.]*

*Sondes possibles :*

- Qu'est-ce que ces animaux *[pointer vers un groupe]* ont en commun ?
- En quoi sont-ils différents de ces animaux ?

**Connaissance et perception de risque (5-10 min)**

Aujourd'hui, nous allons parler de la relation entre les animaux et les *[eleveurs/vendeurs]* comme vous.

**3. Vous avez déjà parlé quelques** **maladies causées par les animaux que vous avez entendu parler. Qu’est-ce que vous savez de ces maladies ?**

*Sondes possibles :*

- Que savez-vous de la façon dont ces maladies se transmettent des animaux aux humains ?
- Que savez-vous de la rage ? *[Demandez seulement si personne n'a pas déjà parlé de la rage.]*

**4. Dans quelle mesure les** *[éleveurs / vendeurs]* **comme vous sont-ils préoccupés par les maladies qui proviennent des animaux ?**

*Sondes possibles :*

- Comment cela se compare-t-il à d'autres soucis dans votre vie ?
- Qu'est-ce qui pourrait rendre un boucher comme vous préoccupée par de telles maladies qui passent d'animaux à l'homme ?

**Comportements de prévention (60-65 min)**

**5. Qu'est-ce qu'on vous a dit que les** *[éleveurs / vendeurs]* **comme vous font pour empêcher les maladies de passer des animaux aux humains ?**

*Sondes possibles :*

- Autres moyens ?
- Qu'en est-il des croyances locales sur la façon de se protéger contre les maladies qui viennent des animaux ?

**6. Maintenant, on va faire une dynamique et organiser ces cartes en groupe. Chaque carte contient l'image d'une action. A chaque action, je leur demanderai si c'est faisable pour les** *[éleveurs / vendeurs]* **comme vous. Ce qui signifie que s’ils trouvent cela facile, ils peuvent le faire, si le matériel nécessaire est facile à obtenir, etc. Ensuite, nous discuterons de la question de savoir si l'action servira à prévenir les maladies causées par les animaux.**

**[***Examinez et discutez chaque pratique en particulier. Lire la description de la première image.*]

| **A** | Gardez les animaux séparés des lieux d’habitation |
| --- | --- |
| **B** | Désinfecter les enclos pour animaux |
| **C** | Évitez les morsures de chien |
| **E** | Vacciner les animaux (comme chiens, poulets, vaches …) |
| **F** | Faire bouillir le lait pendant 30 min avant de boire |
| **I** | Éviter de manger de la viande d’animaux malades |
| **J** | Éviter de manger de la viande de brousse |
| **K** | Couvrir les coupures ou les blessures sur la peau lorsque vous manipulez des animaux |
| **L** | Porter une tenue de protection avant de toucher les carcasses |
| **M** | Enterrer les carcasses d’animaux malades et les fœtus avortés |

*[Après avoir lu la description de la première image, discutez avec le groupe]*

**Que pensez-vous si le comportement est facile à réaliser pour les** *[éleveurs / vendeurs]* **comme vous? C'est-à-dire que, dans le monde actuel, est-ce-que les bouchers le font ?**

*[Vous pouvez donner des exemples de différents aspects de faisabilité qui s'appliquent à l'action en question pour clarifier un peu plus, comme certains des aspects suivants]*

- - Nombre d'étapes nécessaires à la réalisation de l'action
  - Comme il est facile ou difficile de faire les étapes
  - Comme il est facile ou difficile d'organiser ou de planifier l'action (p. ex. prendre des rendez-vous).
  - Le temps nécessaire pour faire l'action (c'est quelque chose que vous n'avez à faire qu'une seule fois ou que vous devez faire et refaire, le temps passé à faire l'action que vous ne faites rien d'autre, etc.)
  - Comme il est facile ou difficile d'obtenir ce dont vous avez besoin pour faire l'action
  - Coût/prix des matériaux (p.ex. savon)
  - La mesure dans laquelle l'action est une norme avec les éleveurs/vendeurs
  - Ce que la culture dicte à propos de l'action

*Sondes possibles :*

- Autres opinions ?
- Par exemple, les éleveurs/vendeurs trouvent-ils cela facile [action X] ?
- Est-ce que les bouchers réalisent de le faire ?

*[Écouter l'opinion de certains membres du groupe sur la faisabilité d'une action. En raison du temps, tout le monde n'a pas à donner son opinion. Calculer 3 minutes par action.]*

**Maintenant, levez la main si vous pensez que, pour les** *[éleveurs / vendeurs]* **comme vous,** *[l’action]***...**

- **est très facile de réaliser**
- **est plus ou moins facile à réaliser**
- **n'est pas facile à réaliser**

*[Ensuite, placez l'action sur le tableau à feuilles et prenez la deuxième image de la même action pour commencer les questions sur l'efficacité. Écoutez l'opinion de certains membres du groupe sur l'efficacité de l'action X. A cause du temps, tout le monde n'a pas besoin de donner son opinion.]*

**Supposons maintenant que** *[nommez quelques défis qu'ils ont mentionnés pour la faisabilité]* **ne soient plus un problème et qu'il soit facile à réaliser le comportement. Que pensez-vous si [l'action X] est utile pour la prévention des zoonoses ?**

*Sondes possibles :*

- Autres opinions ?
- Comment cela fonctionne-t-il ?
- Qu'est-ce qui vous fait croire qu'il est efficace/qu'il fonctionne bien pour prévenir la maladie ?

*[Calculer 3 minutes par action.]*

**Maintenant, levez la main si vous pensez que** *[l’action]* **pour la prévention des maladies zoonoses. Levez la main si vous pensez...**

- **est très utile pour la prévention des maladies zoonoses**
- **est plus ou moins utile pour la prévention des maladies zoonoses**
- **N’est pas utile pour la prévention des maladies zoonoses**

*[Ensuite, placez l'action sur le tableau à feuilles mobiles. Répétez l'opération avec la carte suivante. Après les premières cartes, lorsque le groupe est plus à l'aise avec l'activité, il n'est pas nécessaire d'expliquer chaque groupe de « un peu, plus ou moins et beaucoup ». Vous pouvez dire au groupe : « Maintenant, il faut choisir. Ceux qui pensent un peu, plus ou moins ou beaucoup.»]*

**Hygiène (5 min)**

Maintenant, je voudrais parler de la propreté.

**7. Quand s'agit-il de traiter avec des animaux, quand est-il important de se laver soi-même ?**

*Sondes possibles :*

- Après quelles interactions avec les animaux devez-vous vous laver ?
- Quelles parties de votre corps est-il important de nettoyer après avoir soigné des animaux ou de la viande ?
- Quand vous considérez-vous assez propre ? *[Par exemple, n'a pas l'air sale, odeur, etc.]*

**8. Pensez à l'endroit où vous manipulez de la viande crue : Quand est-il important de laver cette zone ?**

*Sondes possibles :*

- Comment quelqu'un devrait le laver ? *[Produits, processus]*
- Comment décideriez-vous si quelque chose mérite d'être nettoyé ?
- Comment décideriez-vous si c'est assez propre ?

**Chercher de soins (10 min)**

Maintenant, je voudrais parler des cas où les animaux sont malades.

**9. Quels sont les signes ou les symptômes qui pourraient permettre aux** *[éleveurs / vendeurs]* **comme vous de suspecter chez un animal une maladie ?**

**10. Qu’est-ce que font les** *[éleveurs / vendeurs]* **comme vous lorsqu’ils suspectent une maladie chez un de leurs animaux ?**

*Sondes possibles :*

- Pourquoi font-ils cela ? Que pourraient-ils faire d’autre ? Pourquoi ?

**11. Qu'est-ce qui motive les** *[éleveurs / vendeurs]* **comme vous à appeler un vétérinaire ?**

*Sondes possibles :*

- Dans quelles situations ?
- Pourquoi quelqu'un préférerait-il ne pas appeler un vétérinaire ?

**12. Qu'est-ce qui motive les** *[éleveurs / vendeurs]* **comme vous à faire des tests sur les animaux pour voir s’ils sont malades ?**

*Sondes possibles :*

- Pour quelles maladies ? Dans quelles situations ?
- Pourquoi quelqu'un préférerait-il ne pas faire des tests ?

**13. Dans quels types de situations un** *[éleveur / vendeur]* **peut-il tuer les animaux malades ?**

*Sondes possibles :*

- Qu'est-ce qui peut rendre difficile pour une personne de tuer les animaux malades ?
- Que font-ils avec les animaux lorsqu’ils les ont tués ?
- Autres situations ?

On a parlé d'animaux malades. Maintenant, j'aimerais parler du moment où les humains tombent malades.

**14. Quels sont les facteurs qui influencent la recherche de soins de santé pour les** *[éleveurs / vendeurs]* **comme vous ?**

*Sondes possibles :*

- Quels symptômes peuvent inciter quelqu'un à se rendre dans un centre de santé pour un membre de sa famille ?
- Autres facteurs ?

**Sources de communication (10 min)**

Maintenant, j’aimerais maintenant parler un peu de la façon dont les gens de cette communauté obtiennent de l'information sur la santé.

**15. Laquelle de ces sources est la meilleure source d’information sur votre santé et de celle de votre famille ?**

*Sondes possibles :*

- Qu'est-ce qui change quand il s'agit de la santé de vos animaux ?
- **Comment les choses changent-elles en temps de crise ?**

**16. Supposons que vous voyez ou entendez quelques informations sur la santé. Comment décidez-vous si ces informations sont fiables ?**

*Sondes possibles :*

- C'est-à-dire, quelles sont les caractéristiques de l'information qui vous aident à décider si l'information est fiable ?
- Sonder les canaux *[par exemple, radio, télévision, internet, téléphone, réunions communautaires]*, les sources d'information *[leaders communautaires, leaders religieux, voisins, prestataires de soins de santé, guérisseurs traditionnels]*, le contenu de l'information elle-même
- **Comment les choses changent-elles en temps de crise ?**

**17. Selon vous, quels sont les meilleurs moyens pour les autorités sanitaires de communiquer rapidement l'information à** *[éleveurs / vendeurs]* **comme vous en cas de crise ?**

*Sondes possibles :*

- Pendant une crise, quel type d'information serait important pour vous ?
- Quels autres moyens les autorités sanitaires pourraient-ils prendre pour communiquer rapidement ce genre d'information aux *[éleveurs / vendeurs]* comme vous ?

**Conclusion (5 min)**

Merci de m'avoir parlé aujourd'hui. Vous avez partagé *[Résumez ce dont vous avez discuté avec les participants].*

**18. Y a-t-il autre chose que vous aimeriez nous faire savoir sur les** *[éleveurs / vendeurs]* **comme vous et comment ils interagissent avec les animaux ?**

Merci beaucoup pour le temps que vous nous avez consacré.
