# Supplementary material for: Balancing the uncertain and unpredictable nature of possible zoonotic disease transmission with the value placed on animals: Findings from a qualitative study in Guinea
Source: PLOS Glob Public Health. 2024 Mar 28;4(3):e0001174. doi: 10.1371/journal.pgph.0001174 (PMC10977678; doi:10.1371/journal.pgph.0001174)
Supplement: S2 Appendix — (DOCX) [file pgph.0001174.s002.docx]

**Guide Recommandations /directives pour les entretiens approfondis**

**CHEFS LOCAUX**

**Rôle dans la communauté (15-20 min)**

**1. J'aimerais d'abord apprendre à vous connaître un peu**.  *[Veuillez noter le sexe dans vos notes.]*

*Sondes possibles :*

- Âge
- Langue(s) parlée(s)
- Profession
- Nombre d'années dans la profession

**2. Veuillez me parler de votre rôle en tant que chef local.**

*Sondes possibles :*

- Depuis combien de temps assumez-vous ce rôle ?
- Quel est l'éventail des responsabilités que vous assumez en tant que chef local ?
- Type/gamme d'interactions avec les membres de la communauté
- Se faire une idée des autres interactions, responsabilités, etc.

**3. Veuillez parler d'une journée typique que vous pourriez avoir en tant que leader local.**

*Sondes possibles :*

- Type de réunions/événements
- Type d'interactions avec les gens
- Type de personnes
- Heures de la journée

**4. Veuillez décrire des moments dans vos activités comme leader local où vous pourriez avoir des interactions avec les animaux.**

*Sondes possibles :*

- Veuillez décrire le type d'interaction. A quelle occasion *[événement ou situation]* ?
- Quel type d'animal ?
- Quelles sont les personnes qui pourraient également être présentes et combien d'entre elles ?

**5. Que savez-vous de la façon dont les membres de votre communauté gèrent leurs animaux?**

*Sondes possibles :*

- Ce qui motive la gestion des animaux
- Quelles sont les contraintes auxquelles font face les gens de votre communauté en ce qui concerne la gestion des animaux ?
- Qui influence les décisions concernant le traitement des maladies des animaux dans la communauté ?

**Connaissance et perception du risque (20-25 min)**

Comme je l'ai déjà dit, nous voulons comprendre les expériences et les opinions des communautés au sujet des maladies causées par les animaux. Vous avez déjà parlé des maladies causées par les animaux que vous avez spécifiquement entendu parler.

**6. Maintenant, veuillez expliquer ce que vous savez de ces maladies.**

*Sondes possibles :*

- Que savez-vous de la façon dont ces maladies se transmettent des animaux aux humains? Que savez-vous de la rage ?

**7. À quel point êtes-vous, vous-même, préoccupé par la possibilité de contracter des maladies transmises par les animaux ? Qu'est-ce qui vous préoccupe ? Veuillez expliquer.**

*Sondes possibles :*

- Qu'est-ce qui vous préoccupe au sujet de ces maladies ?
- Quelle maladie vous soucient le plus ? Qu'est-ce qui vous inquiète le plus ?
- Que savez-vous de ce qui peut arriver à quelqu'un qui est infecté par ces maladies ?

**8.** **Maintenant que j'ai une idée de ce que vous ressentez à l'égard de ces types de maladies, j'aimerais en savoir un peu plus sur ce que vous pensez que les gens de votre communauté croient.** *[Explorez les deux scénarios.]*

1. Admettons qu’il y avait une épidémie de maladie contagieuse et que les autorités demandaient aux gens de votre communauté de rester chez eux et d'éviter les grands attroupements. Que pensez-vous que la population ferait d’une telle demande des autorités ?
2. Admettons maintenant qu'il y avait une épidémie qui est transmise par les poules aux humains et que les populations sont en train de tomber malades. Les autorités sanitaires régionales demandaient aux gens de votre communauté de dépister leurs volailles et d'abattre les animaux infectés.

*Sondes possibles :*

- Qu'est-ce qui les empêcherait de suivre la directive des autorités ?
- Selon vous, quel niveau de risque la communauté percevrait-elle avec une telle directive des autorités ?
- Dans quelle mesure feront-ils confiance à ce que disent les autorités ?

**Sources et canaux d’information (15 min)**

**9. Parlons maintenant de l'endroit où vous pouvez obtenir de l'information que vous aimeriez peut-être partager avec votre communauté sur la santé.**

**D’où obtenez-vous des informations sur la santé de la population locale ?**

**D’où obtenez-vous des informations sur la santé des animaux ?**

*Sondes possibles :* *Vous pouvez poser des questions sur des canaux de communication spécifiques tels que*

- la radio
- la télévision
- l’internet
- les médias sociaux
- les chefs religieux
- les prestataires de santé
- les guérisseurs traditionnels

**10. Comment décidez-vous si l'information est fiable ou non ?**

*Sondes possibles :*

- source d’information
- canal de diffusion de l'information
- contenu de l'information
- crédibilité

**11. Dans le cas d'une épidémie, d’où obtenez-vous des informations ?**

*Sondes possibles :*

- Quelle est l’efficacité de ce système de communication ?
- Quels sont les autres moyens de communication dont disposent le gouvernement et les autorités sanitaires pour informer la population ?
- Quel est le moyen de communication préférable pour vous ? Pourquoi ?
- Comment décidez-vous si l'information est fiable ou non ?

**Rôle pendant l’épidémie (30 min)**

**12. Comment décririez-vous votre rôle pendant une épidémie chez les animaux et les humains ?**

*Sondes possibles :*

- Quelles sont vos autres responsabilités en cas d’épidémie dans la/les communauté(s) que vous servez ?
- Jusqu’à quel point vous sentez-vous préparé pour assumer votre rôle pendant une épidémie ? Comment ?
- Jusqu’à quel point pensez-vous que la communauté aura confiance en vous lors d’une épidémie ? Pourquoi ?
- Qu’est-ce qui pourrait vous permettre de vous sentir plus équipé ou soutenu pour réagir face à une épidémie ?

**13. Veuillez me dire d'un récent événement zoonotique ou vous avez joué un rôle en aidant votre communauté à rester informée et à répondre efficacement**

*Sondes possibles :*

- Qu’est-ce-que qui s’est passé en général ?
- Comment avez-vous vérifié si l'information que vous avez entendue était vraie ou non ?
- Quelle a été la première chose que vous avez faite ? Qu’avez-vous fait alors ?
- Quel type de plan, le cas échéant, avez-vous lancé ? Qui d'autre avez-vous impliqué dans vos décisions sur ce qu'il faut faire ?
- Quel rôle avez-vous joué dans la communication de l'information à la communauté ? Comment leur avez-vous parlé de l'événement et de ce qu'ils devraient faire pour se protéger et protéger leur famille ? Qui avez-vous informé ? Comment ont-ils réagi ?
- Quel rôle avez-vous joué dans la communication ou le soutien des informations envoyées par les autorités ? Comment les gens dans votre communauté ont-ils réagi ?

*[S'ils décrivent leur rôle dans un petit événement, comme un cas de rage, posez-lui les questions suivantes.]*

Et une épidémie plus généralisée comme celle d'Ebola ?

*Sondes possibles :*

- Qui s’est passé en général ?
- Comment avez-vous vérifié si l'information que vous avez entendue était vraie ou non ?
- Quelle a été la première chose que vous avez faite ? Qu’avez-vous fait alors ?
- Quel type de plan, le cas échéant, avez-vous lancé ? Qui d'autre avez-vous impliqué dans vos décisions sur ce qu'il faut faire ?
- Quel rôle avez-vous joué dans la communication de l'information à la communauté ? Comment leur avez-vous parlé de l'événement et de ce qu'ils devraient faire pour se protéger et protéger leur famille ? Qui avez-vous informé ? Comment ont-ils réagi ?
- Quel rôle avez-vous joué dans la communication ou le soutien des informations envoyées par les autorités ? Comment les gens dans votre communauté ont-ils réagi ?

*[Si la personne n'a pas d'expérience récente, posez-lui les questions suivantes.]*

Nous avons parlé plus tôt de ce que vous pensez que les gens de votre communauté feraient si, pendant une épidémie, les autorités disaient aux gens de rester chez eux et d'éviter les foules nombreuses.

Quel rôle pensez-vous que vous auriez dans une telle situation ?

*Sondes possibles :*

- - A votre niveau que pourriez-vous faire pour aider les gens à respecter les directives des autorités ?
  - Comment pensez-vous que les gens de votre communauté réagiraient si vous souteniez directives des autorités ?

**Conclusion (5 min)**

Merci de m'avoir parlé aujourd'hui. Vous avez partagé *[Résumez ce dont vous avez discuté avec le répondant pendant l'entrevue]* avec moi.

**14. Y a-t-il d’autres suggestions que vous aimeriez faire sur la manière dont les autorités locales, régionales et nationales pourraient prévenir et se préparer pour les épidémies de maladies transmises par les animaux ?**

**15. Y a-t-il autre chose que vous aimeriez nous faire savoir sur votre communauté et les maladies qui sont transmises par les animaux ?**

Merci beaucoup pour le temps que vous nous avez consacré.

**Guide Recommandations /directives pour les entretiens approfondis (EI)**

**PROFESSIONNELLES DES MEDIAS**

**Rôle dans la communauté (15-20 min)**

**1. J'aimerais d'abord apprendre à vous connaître un peu**.  *[Veuillez noter le sexe dans vos notes.]*

*Sondes possibles :*

- Âge
- Langue(s) parlée(s)
- Profession
- Nombre d'années dans la profession

**2. Veuillez me parler de votre rôle en tant qu’un professionnel de media.**

*Sondes possibles :*

- Quel est l'éventail des responsabilités que vous assumez ?
- Type/gamme d'interactions avec les membres de la communauté
- Se faire une idée des autres interactions, responsabilités, etc.

**3. Veuillez parler d'une journée typique que vous pourriez avoir en tant que professionnel de media.**

*Sondes possibles :*

- Type de réunions/événements
- Type d'interactions avec les gens
- Type de personnes
- Heures de la journée

**4. Dans la communauté où vous travaillez comme un agent de média, que savez-vous de la façon dont les gens gèrent leurs animaux ?**

*Sondes possibles :*

- Ce qui motive la gestion des animaux ? Quelles sont les contraintes auxquelles font face les gens de votre communauté en ce qui concerne la gestion des animaux ?
- Qui influence les décisions concernant le traitement des maladies des animaux dans la communauté ?

**Connaissance et perception du risque (20-25 min)**

Comme je l'ai déjà dit, nous voulons comprendre les expériences et les opinions des communautés au sujet des maladies causées par les animaux. Vous avez déjà parlé des maladies causées par les animaux que vous avez spécifiquement entendu parler.

**5. Maintenant, veuillez expliquer ce que vous savez de ces maladies.**

*Sondes possibles :*

- Que savez-vous de la façon dont ces maladies se transmettent des animaux aux humains? Que savez-vous de la rage ?

**6. À quel point êtes-vous, vous-même, préoccupé par la possibilité de contracter des maladies transmises par les animaux ? Qu'est-ce qui vous préoccupe ? Veuillez expliquer.**

*Sondes possibles :*

- Qu'est-ce qui vous préoccupe au sujet de ces maladies ?
- Quelle maladie vous soucient le plus ? Qu'est-ce qui vous inquiète le plus ?
- Que savez-vous de ce qui peut arriver à quelqu'un qui est infecté par ces maladies ?

**7.** **Maintenant que j'ai une idée de ce que vous ressentez à l'égard de ces types de maladies, j'aimerais en savoir un peu plus sur ce que vous pensez que les gens de votre communauté croient.** *[Explorez les deux scénarios.]*

1. Admettons qu’il y avait une épidémie de maladie contagieuse et que les autorités demandaient aux gens de votre communauté de rester chez eux et d'éviter les grands attroupements. Que pensez-vous que la population ferait d’une telle demande des autorités ?
2. Admettons maintenant qu'il y avait une épidémie qui est transmise par les poules aux humains et que les populations sont en train de tomber malades. Les autorités sanitaires régionales demandaient aux gens de votre communauté de dépister leurs volailles et d'abattre les animaux infectés.

*Sondes possibles :*

- Selon vous, quel niveau de risque la communauté percevrait-elle avec une telle directive des autorités ?
- Dans quelle mesure feront-ils confiance à ce que disent les autorités ?

**Sources et canaux d’information (15 min)**

**8. Parlons maintenant de l'endroit où vous pouvez obtenir de l'information que vous aimeriez diffuser sur la santé.**

**D’où obtenez-vous des informations sur la santé de la population locale ?**

**D’où obtenez-vous des informations sur la santé des animaux ?**

*Sondes possibles :* *Vous pouvez poser des questions sur des canaux de communication spécifiques tels que*

- la radio
- la télévision
- l’internet
- les médias sociaux
- les chefs religieux
- les prestataires de santé
- les guérisseurs traditionnels

**9. En tant que professionnel des médias, comment vous tenez-vous informé de ce qui se passe dans les communautés ?**

*Sondes possibles :*

- Par quels autres moyens vos collègues pourraient-ils s’informer de ce qui se passe dans les communautés ?
- Si les gens de la communauté locale veulent communiquer avec des agents des médias comme vous au sujet de questions de santé, de quelle façon pourraient-ils le faire ?

**10. Dans le cas d'une épidémie, d’où obtenez-vous des informations ?**

*Sondes possibles :*

- Quelle est l’efficacité de ce système de communication ?
- Quels sont les autres moyens de communication dont disposent les autorités sanitaires pour informer la population ?
- Quel est le moyen de communication préférable pour vous ? Pourquoi ?
- Comment décidez-vous si l'information est fiable ou non ?

**Rôle pendant l’épidémie (30 min)**

**11. Comment décririez-vous votre rôle pendant une épidémie chez les animaux et les humains ?**

*Sondes possibles :*

- Quelles sont vos autres responsabilités en cas d’épidémie ?
- Jusqu’à quel point vous sentez-vous préparé pour assumer votre rôle pendant une épidémie ? Comment ?
- Jusqu’à quel point pensez-vous que la communauté aura confiance dans les agents des médias comme vous lors d’une épidémie ? Pourquoi ?
- Qu’est-ce qui pourrait vous permettre de vous sentir plus équipé ou soutenu pour réagir face à une épidémie ?

**12. Imaginez que vous voyez sur les réseaux sociaux un message concernant une maladie qui tue** *[Choisissez selon le profil de région : le bétail ou la volaille]* **dans un certain village.**

*Sondes possibles :*

- Quelle serait votre première réaction ?
- Si vous décidez de faire une investigation plus approfondie, comment vérifieriez-vous l'information ?
- Que feriez-vous si vous vous rendiez compte que l'information n'est pas vraie ?

**13. Veuillez me dire d'une épidémie** **zoonotique ou vous avez joué un rôle en aidant votre communauté à rester informée et à répondre efficacement**

*Sondes possibles :*

- Qu’est-ce-que qui s’est passé en général ?
- Comment avez-vous vérifié si l'information que vous avez entendue était vraie ou non ?
- Quelle a été la première chose que vous avez faite ? Qu’avez-vous fait alors ?
- Quel rôle avez-vous joué dans la communication de l'information à la communauté ? Comment leur avez-vous parlé de l'événement et de ce qu'ils devraient faire pour se protéger et protéger leur famille ? Qui avez-vous informé ? Comment ont-ils réagi ?
- Quel rôle avez-vous joué dans la communication ou le soutien des informations envoyées par les autorités ? Comment les gens dans votre communauté ont-ils réagi ?

*[Si la personne n'a pas d'expérience récente, posez-lui les questions suivantes.]*

Nous avons parlé plus tôt de ce que vous pensez que les gens de votre communauté feraient si, pendant une épidémie, les autorités disaient aux gens de rester chez eux et d'éviter les foules nombreuses.

Quel rôle pensez-vous que vous auriez dans une telle situation ?

*Sondes possibles :*

- - A votre niveau que pourriez-vous faire pour aider les gens à respecter les directives des autorités ?
  - Comment pensez-vous que les gens de votre communauté réagiraient si vous souteniez directives des autorités ?

**Conclusion (5 min)**

Merci de m'avoir parlé aujourd'hui. Vous avez partagé *[Résumez ce dont vous avez discuté avec le répondant pendant l'entrevue]* avec moi.

**14. Y a-t-il d’autres suggestions que vous aimeriez faire sur la manière dont les autorités locales, régionales et nationales pourraient prévenir et se préparer pour les épidémies de maladies transmises par les animaux ?**

**15. Y a-t-il autre chose que vous aimeriez nous faire savoir sur votre communauté et les maladies qui sont transmises par les animaux ?**

Merci beaucoup pour le temps que vous nous avez consacré.

**Guide Recommandations /directives pour les entretiens approfondis**

**PRESTATAIRES**

**Rôle dans la communauté (15-20 min)**

**1. J'aimerais d'abord apprendre à vous connaître un peu**.  *[Veuillez noter le sexe dans vos notes.]*

*Sondes possibles :*

- Âge
- Langue(s) parlée(s)
- Profession
- Nombre d'années dans la profession

**2. Veuillez me parler de votre rôle en tant que prestataire.**

*Sondes possibles :*

- Quel est l'éventail des responsabilités que vous assumez en tant que prestataire ?
- Type/gamme d'interactions avec les membres de la communauté
- Se faire une idée des autres interactions, responsabilités, etc.

**3. S'il vous plaît, parlez-moi d'une journée typique que vous pourriez avoir en tant que prestataire.**

*Sondes possibles :*

- Type de clients/patients
- Type d'interactions avec les gens
- Heures de la journée

**Connaissance et perception du risque (20-25 min)**

Comme je l'ai déjà dit, nous voulons comprendre les expériences et les opinions des communautés au sujet des maladies causées par les animaux.

**4. Vous avez déjà parlé des maladies causées par les animaux que vous avez entendu parler. J'aimerais maintenant vous parler de ce que vous savez de ces maladies.**

*Sondes possibles :*

- Que savez-vous de la façon dont ces maladies se transmettent des animaux aux humains ?
- Que savez-vous de la rage ?

**5. Veuillez décrire des moments dans vos activités comme prestataire où vous faites face à des maladies que viennent des animaux.**

*Sondes possibles :*

- Veuillez décrire le type maladie.
- Quel est l'événement ou la situation ?
- A quel moment de la maladie vous avez tendance à voir la personne
- *[Si ne mentione pas la rage…]* De quelle façon et à quelle fréquence votre travail interagit-il avec la rage ?

**6. À quel point êtes-vous, vous-même, préoccupé par la possibilité de contracter des maladies transmises par les animaux ? Qu'est-ce qui vous préoccupe ? Veuillez expliquer.** *[Si quelqu'un parle d'une des maladies en particulier, utilisez ces sondes, mais en mentionnant spécifiquement cette maladie.]*

*Sondes possibles :*

- Qu'est-ce qui vous préoccupe au sujet de ces maladies ?
- Quelle maladie vous soucient le plus ? Qu'est-ce qui vous inquiète le plus ?
- Que savez-vous de ce qui peut arriver à quelqu'un qui est infecté par ces maladies ?

**Sources et canaux d’information (15 min)**

**7. Parlons maintenant de l'endroit où vous pouvez obtenir de l'information.**

**D’où obtenez-vous des informations sur la santé de la population locale ?**

**D’où obtenez-vous des informations sur la santé des animaux ?**

*Sondes possibles :* *Vous pouvez poser des questions sur des canaux de communication spécifiques tels que*

- la radio
- la télévision
- l’internet
- les médias sociaux
- les chefs religieux
- les prestataires de santé
- les guérisseurs traditionnels

**8. Comment décidez-vous si l'information est fiable ou non ?**

*Sondes possibles :*

- source d’information
- canal de diffusion de l'information
- contenu de l'information
- crédibilité

**9. En tant que prestataire de santé, comment vous tenez-vous informé de ce qui se passe dans les communautés ?**

*Sondes possibles :*

- Par quels autres moyens vos collègues pourraient-ils s’informer de ce qui se passe dans les communautés ?
- Si les gens de la communauté locale veulent communiquer avec des prestataires comme vous au sujet de questions de santé, de quelle façon pourraient-ils le faire ?

**10. Dans le cas d'une épidémie, d’où obtenez-vous des informations ?**

*Sondes possibles :*

- Quelle est l’efficacité de ce système de communication ?
- Quels sont les autres moyens de communication dont disposent le gouvernement et les autorités sanitaires pour informer la population ?
- Quel est le moyen de communication préférable pour vous ? Pourquoi ?
- Comment décidez-vous si l'information est fiable ou non ?

**Rôle pendant l’épidémie (30 min)**

**11. Comment décririez-vous votre rôle pendant une épidémie chez les humains ?**

*Sondes possibles :*

- Quelles sont vos autres responsabilités en cas d’épidémie dans la/les communauté(s) que vous servez ?
- Jusqu’à quel point vous sentez-vous préparé pour assumer votre rôle pendant une épidémie ? Comment ?
- Qu’est-ce qui pourrait vous permettre de vous sentir plus équipé ou soutenu pour réagir face à une épidémie ?

**12. Veuillez me dire d'un récent événement zoonotique ou vous avez joué un rôle en aidant votre communauté.**

*Sondes possibles :*

- Qu’est-ce-que qui s’est passé en général ?
- Quel rôle avez-vous joué dans la communication de l'information à la communauté ?

*[S'ils décrivent leur rôle dans un petit événement, comme un cas de rage, posez-lui les questions suivantes.]*

Et une épidémie plus généralisée ?

*Sondes possibles :*

- Qu’est-ce-que qui s’est passé en général ?
- Comment avez-vous vérifié si l'information que vous avez entendue était vraie ou non ?
- Quelle a été la première chose que vous avez faite ? Qu’avez-vous fait alors ?
- Quel rôle avez-vous joué dans la communication de l'information à la communauté ? Comment leur avez-vous parlé de l'événement et de ce qu'ils devraient faire pour se protéger et protéger leur famille ? Qui avez-vous informé ? Comment ont-ils réagi ?
- Qu'avez-vous observé au sujet de la relation entre les communautés et les fournisseurs de soins de santé à l'époque ?
- Quel rôle avez-vous joué dans la communication ou le soutien des informations envoyées par les autorités ? Comment les gens dans votre communauté ont-ils réagi ?

*[Si la personne n'a pas d'expérience récente, posez-lui les questions suivantes.]*

Imaginez que vous avez vu plusieurs cas de personnes atteintes d'une maladie d'origine animale, comme la grippe aviaire. Qu'est-ce que vous feriez ?

*Sondes possibles :*

- Quelle est la première chose que vous feriez ? Et après ?
- Qui informeriez-vous ? Pourquoi ? Comment les informeriez-vous ?
- Quel rôle feriez-vous joué dans la communication ou le soutien des informations envoyées par les autorités ?

**Conclusion (5 min)**

Merci de m'avoir parlé aujourd'hui. Vous avez partagé *[Résumez ce dont vous avez discuté avec le répondant pendant l'entrevue]* avec moi.

**13. Y a-t-il d’autres suggestions que vous aimeriez faire sur la manière dont les autorités locales, régionales et nationales pourraient prévenir et se préparer pour les épidémies de maladies transmises par les animaux ?**

**14. Y a-t-il autre chose que vous aimeriez nous faire savoir sur votre communauté et les maladies qui sont transmises par les animaux ?**

Merci beaucoup pour le temps que vous nous avez consacré.

**Guide Recommandations /directives pour les entretiens approfondis**

**VÉTÉRINAIRE**

**Rôle dans la communauté (15-20 min)**

**1. J'aimerais d'abord apprendre à vous connaître un peu**.  *[Veuillez noter le sexe dans vos notes.]*

*Sondes possibles :*

- Âge
- Langue(s) parlée(s)
- Profession
- Nombre d'années dans la profession

**2. Veuillez me parler de votre rôle en tant que vétérinaire.**

*Sondes possibles :*

- Quel est l'éventail des responsabilités que vous assumez en tant que vétérinaire ?
- Type/gamme d'interactions avec les membres de la communauté
- Se faire une idée des autres interactions, responsabilités, etc.

**3. S'il vous plaît, parlez-moi d'une journée typique que vous pourriez avoir en tant que vétérinaire.**

*Sondes possibles :*

- Type de animaux
- Type d'interactions avec les animaux
- Type d'interactions avec les gens
- Heures de la journée

**4. Que savez-vous de la façon dont les membres de votre communauté gèrent leurs animaux ?**

*Sondes possibles :*

- Ce qui motive la gestion des animaux
- Quelles sont les contraintes auxquelles font face les gens de votre communauté en ce qui concerne la gestion des animaux ?
- Qui influence les décisions concernant le traitement des maladies des animaux dans la communauté ?

**Connaissance et perception du risque (20-25 min)**

Comme je l'ai déjà dit, nous voulons comprendre les expériences et les opinions des communautés au sujet des maladies causées par les animaux.

**5. Vous avez déjà parlé des maladies causées par les animaux que vous avez entendu parler. J'aimerais maintenant vous parler de ce que vous savez de ces maladies.**

*Sondes possibles :*

- Que savez-vous de la façon dont ces maladies se transmettent des animaux aux humains ?
- Que savez-vous de la rage ? *[Demandez seulement si la personne n'a pas déjà parlé de la rage.]*

**6. Dans le cadre de votre travail, dans quelle mesure voyez-vous des maladies qui peuvent passer de l'animal à l'homme ?**

*Sondes possibles :*

- Veuillez décrire les types les plus courants de zoonoses que vous voyez.
- A quel moment de la maladie vous avez tendance à voir l’animal ?
- *[Si ne mentione pas la rage…]* De quelle façon et à quelle fréquence votre travail interagit-il avec la rage ?

**7. À quel point êtes-vous, vous-même, préoccupé par la possibilité de contracter des maladies transmises par les animaux ? Qu'est-ce qui vous préoccupe ? Veuillez expliquer.**

*Sondes possibles :*

- Qu'est-ce qui vous préoccupe au sujet de ces maladies ?
- Quelle maladie vous soucient le plus ? Qu'est-ce qui vous inquiète le plus ?
- Que savez-vous de ce qui peut arriver à quelqu'un qui est infecté par ces maladies ?

**8. Imaginez qu'il y avait une épidémie qui est transmise par les poules aux humains et que les populations sont en train de tomber malades. Les autorités sanitaires régionales demandaient aux gens de votre communauté de dépister leurs volailles et d'abattre les animaux infectés.**

*Sondes possibles :*

- Que penserait la communauté de cette directive ?
- Dans quelle mesure feront-ils confiance à ce que disent les autorités ?
- Dans quelle mesure le respecteraient-ils, à votre avis ?
- Qu'est-ce qui les empêcherait de suivre la directive des autorités ?
- Selon vous, quel niveau de risque la communauté percevrait-elle avec une telle directive des autorités ?

**Sources et canaux d’information (15 min)**

**9. Parlons maintenant de l'endroit où vous pouvez obtenir de l'information.**

**D’où obtenez-vous des informations sur la santé de la population locale ?**

**D’où obtenez-vous des informations sur la santé des animaux ?**

*Sondes possibles :* *Vous pouvez poser des questions sur des canaux de communication spécifiques tels que*

- la radio
- la télévision
- l’internet
- les médias sociaux
- les chefs religieux
- les prestataires de santé
- les guérisseurs traditionnels

**10. Comment décidez-vous si l'information est fiable ou non ?**

*Sondes possibles :*

- source d’information
- canal de diffusion de l'information
- contenu de l'information
- crédibilité

**11. En tant que vétérinaire, comment vous tenez-vous informé de ce qui se passe dans les communautés ?**

*Sondes possibles :*

- Par quels autres moyens vos collègues pourraient-ils s’informer de ce qui se passe dans les communautés ?
- Si les gens de la communauté locale veulent communiquer avec des vétérinaires comme vous au sujet de questions de santé, de quelle façon pourraient-ils le faire ?

**12. Dans le cas d'une épidémie, d’où obtenez-vous des informations ?**

*Sondes possibles :*

- Quelle est l’efficacité de ce système de communication ?
- Quels sont les autres moyens de communication dont disposent le gouvernement et les autorités sanitaires pour informer la population ?
- Quel est le moyen de communication préférable pour vous ? Pourquoi ?
- Comment décidez-vous si l'information est fiable ou non ?

**Rôle pendant l’épidémie (30 min)**

**13. Comment décririez-vous votre rôle pendant une épidémie chez les animaux ?**

*Sondes possibles :*

- Quelles sont vos autres responsabilités en cas d’épidémie dans la/les communauté(s) que vous servez ?
- Jusqu’à quel point vous sentez-vous préparé pour assumer votre rôle pendant une épidémie ? Comment ?
- Qu’est-ce qui pourrait vous permettre de vous sentir plus équipé ou soutenu pour réagir face à une épidémie ?
- Qu'en est-il d'une épidémie de zoonose où de nombreuses personnes tombent malades ? En quoi cela change-t-il votre rôle ?

**14. Veuillez me dire d'un récent événement zoonotique ou vous avez joué un rôle en aidant votre communauté** **à rester informée et à répondre efficacement.**

*Sondes possibles :*

- Qu’est-ce-que qui s’est passé en général ?
- Qu’avez-vous fait alors ?
- Comment les gens ont-ils réagi ?
- Quel rôle avez-vous joué dans la communication de l'information à la communauté ?

*[S'ils décrivent leur rôle dans un petit événement, comme un cas de rage, posez-lui les questions suivantes.]*

Et une épidémie plus généralisée ?

*Sondes possibles :*

- Qu’est-ce-que qui s’est passé en général ?
- Comment avez-vous vérifié si l'information que vous avez entendue était vraie ou non ?
- Quelle a été la première chose que vous avez faite ? Qu’avez-vous fait alors ?
- Quel rôle avez-vous joué dans la communication ou le soutien des informations envoyées par les autorités ? Comment les gens dans votre communauté ont-ils réagi ?

*[Si la personne n'a pas d'expérience récente, posez-lui les questions suivantes.]*

Imaginez qu'un membre de la communauté vous demande de venir soigner une poule malade. Lorsque vous partez, vous découvrez que plusieurs autres poules sont mortes récemment.

*Sondes possibles :*

- Quelle est la première chose que vous feriez ? Et après ? *[Cherchez à approfondir tous les aspects de la répons*e]
- Qui informeriez-vous ? Pourquoi ? Comment les informeriez-vous ?
- Quel rôle feriez-vous joué dans la communication ou le soutien des informations envoyées par les autorités ?

**Conclusion (5 min)**

Merci de m'avoir parlé aujourd'hui. Vous avez partagé *[Résumez ce dont vous avez discuté avec le répondant pendant l'entrevue]* avec moi.

**15. Y a-t-il d’autres suggestions que vous aimeriez faire sur la manière dont les autorités locales, régionales et nationales pourraient prévenir et se préparer pour les épidémies de maladies transmises par les animaux ?**

**16. Y a-t-il autre chose que vous aimeriez nous faire savoir sur votre communauté et les maladies qui sont transmises par les animaux ?**

Merci beaucoup pour le temps que vous nous avez consacré.
