# Supplementary material for: Balancing the uncertain and unpredictable nature of possible zoonotic disease transmission with the value placed on animals: Findings from a qualitative study in Guinea
Source: PLOS Glob Public Health. 2024 Mar 28;4(3):e0001174. doi: 10.1371/journal.pgph.0001174 (PMC10977678; doi:10.1371/journal.pgph.0001174)
Supplement: S1 Data — (DOCX) [file pgph.0001174.s003.docx]

**Legend**

P = Participant

E = Researcher

Document = Source document from where the data originates

**In Document:**
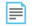
 52 NZ_GD_MA2_2609_T

P5 : Nous sommes de la localité, on sait que c’est très bon pour notre santé mais on fait par manque de temps ; on dit que c’est facile à faire quand tu dis à nos parents d’ici qu’il faut qui bouis le lait au feu après 30 minutes avant le consommer ils vont dire que si c’est toi qui consomme ou eux là on parle du lait de la vache on ignore sa provenance et les conditions de son extraction donc très souvent on le lait par le fait de la routine

**In Document:**
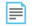
 51 NZ_GD_MA2_2509_T

Il y a certains bouchers qui abattent certains animaux pour revendre or qu’ils savent bien que cet animal ne peut plus vivre pour longtemps mais cela ne t’empêche de l’égorger. Toi consommateur qui ignore tout, vient payer ce genre de viande et la consommer. C’est tout ce que je peux vous dire et je vous encourage de continuer.

Si on savait que ça allait se passer comme ça on aurait pu venir plus nombreux que ça.

**In Document:**
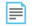
 21 NZ_GD_FU_2509_T

P7 : Moi je dirai que je suis inquiète par ce que les gens élèvent les chiens juste pour le faire ; ils ne s’occupent pas d’eux, ils ne les vaccinent pas et ces chiens sont abandonnés en eux même, ils ne sont ni attacher et s’il arrive à te mordre sa devient compliquer, c’est pourquoi moi je voudrais lancer un appel à tous ceux qui ont des chiens de les faire vacciner

**In Document:**
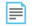
 63 NZ_GD_FR_2709_T

E : Mais quand est-il de lait industriel ?

P6 : J’ai fait un bon temps au fouta, on extrait le lait et on garde pendant 2 jours et on consomme, je n’ai jamais un peulh ou bien voir une femme peulh faire bouillir le lait.

P10 : On chauffe de l’eau et on met dans le thermo-Che et le matin on transfert dans les verres, on met du lait et on fait remué avec le sucre on prend. Ça on fait bouillir à 100 degré.

E : P1 est en train de nous dire au moins qu’elle connait beaucoup de chose dans cette histoire ? Pour vous est ce que c’est très facile ?

P1 : Moi, je n’ai jamais vu quelqu’un faire bouillir le lait de vache, les autres laits, ce que ma fille a dit on fait ça.

**In Document:**
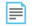
 51 NZ_GD_MA2_2509_T

P4 : Ce n’est pas facile quand ton animal tombe malade et tu l’as payé à 1000fg et c’est tout ton capital ça, donc dire de jeter cela n’est pas du tout facile. C’est malgré nous ‘le propriétaire’ qu’on jette les animaux ici. Cela m’est arrivé une fois pendant ce temps-là je grillais les viandes pour revendre, on m’a revendu une viande qui est déjà mordue par le chien et le vétérinaire m’a dit de ne pas revendre cette viande et j’ai répondu pourquoi cela car j’ai déjà investi mon argent là-dedans et il a insisté de l’enterrer j’ai dit non que je vais l’emmener à la maison, il m’a conseillé que ma famille ne devait pas manger, j’ai expliqué que parce que les chiens capturent des animaux pour nous à la chasse et on mange cela et pourquoi pas une chèvre ?

Donc au final j’avais réfléchi et je l’ai dit si je l’emmène ça et que la famille soit contaminée je suis responsable. J’ai accepté et fait ce qu’avait le vétérinaire.

Si non te débarrasser de lui comme ça ce n’est pas facile, car même les personnes à qui on a acheté cet animal ne nous rembourseront pas notre argent.

**In Document:**
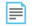
 7 NZ_EN_LC_2609_T

E : voilà ! bon, maintenant, ça c’est qu’ils vont le faire. Maintenant on demande qu’est qui peut alors empêcher la population de suivre les directives là ? qu’est qui pourrai les empêcher de le faire ?

P : ceux qui les empêches de le faire, c’est le problème d’alimentation et le problème de moyen.

E : ok ! parce qu’ils n’ont pas les moyens.

P : voilà ! parce que il y’a d’autres c’est dans ça ils peuvent se nourrir,

E : ok !

P : y’a d’autres aussi quand ils vendent ça, ils paient les condiments, ils paient les nourritures.

E : ok !

P : C’est ce qui les empêches.

E : c’est le problème de moyen qui les empêches d’accepter une telle demande des autorités.

**In Document:**
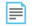
 52 NZ_GD_MA2_2609_T

E : L’IMAGE I EVITER DE MANGER DE LA VIANDE DES ANIMAUX MALADE

EST-CE FACILE A REALISER OU PAS ?

P10 : ce n’est pas facile de faire surtout quand on trouve un animal mort dans le village de ne pas consommer sa viande et souvent on considère même les questions d’hygiène sanitaire de l’animal

P5 : ce n’est pas chose facile à réaliser par exemple dans le village quand un bœuf ou un mouton meurt même après qu’on l’a jeté les enfants peuvent se cacher pour aller prendre ces viandes là et le consommé

P3 : Ce n’est pas facile dans le village parce qu’il y’a certaines familles qui peuvent faire un à deux mois sans manger la viande et les enfants sont en manquent de protéine et si c’est pareil occasion se présente ils ne manqueront pas de se faire plaisir

P1 : Comme ils l’ont si bien mes frères ce n’est pas du tout facile même si on sait que l’animal est malade ils peuvent le prendre s’enfuir dans la brousse et le consommé donc il est très difficile de comprendre, ils ne peuvent pas comprendre qu’on le fait pour leurs santés ; les enfants peuvent prendre l’animal et s’enfuir dans la brousse pour le consommer vous savez il y’a de la protéine

**In Document:**
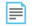
 2 NZ_GD_HU_2509_T

P5 : Pour moi ce n’est pas facile à réalise. Parce que si nous constatons un peu le faire dans les préfectures qui sont un peu développés, mais dans les sous-préfectures on constate également que les gens n’aiment pas envoyer les animaux hors de leur famille puisque vu le danger des voleurs aussi, il y’a des animaux qui leurs appartient pas donc ç ne serait pas important d’éloigner ces animaux de la famille. Dans nos communautés ici par exemple prenons le cas des chiens, c’est pas d’éloigner les chiens….

P9 : Pour moi c’est facile.

**In Document:**
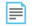
 1 KA_GD_MA2_2609_T.docx

morsure de chien, c’est difficile ou pas ?

P7 : c’est difficile. Parce que chez nous ici les chiens sont laissés à eux-mêmes, ils ne sont pas gardés. Même si toi tu gardes pour toi, mais si tu sors dans la ville, tu croiseras un autre qui est délaissé par son propriétaire. Sur ce plan c’est difficile de se préserver soi-même.

P6 : bon la garde du chien c’est très difficile. Les animaux dont on parle, nos jeunes qui sont derrières les troupeaux, ils vivent avec les chiens en brousse. Ils chassent et font tout. Hors mis la rage même du chien, le chien a un microbe, si ce microbe touche la viande, si tu manges, cela sera le facteur de transmission du microbe chez toi. Donc nous éloigner du chien, c’est difficile pour nous, c’est vraiment difficile.

**In Document:**
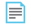
 29 KA_EN_VE_2409_T

E : qu’est-ce qui vous inquiète de plus ?

P : mon inquiétude, est que si on ne vient pas à temps, pour, au moins, lutter contre cette maladie, ça risque de faire beaucoup de dégâts. Par ce que quand vous voyez un chien… je vous donne un exemple, y a un chien qui est venu cette année, un chien qui est venu de la préfecture de Beyla, dans la sous-préfecture de dioula, le gars à envoyer le chien et le chien

(Une visiteuse qui salue en langue malinké)

E : ouhoun

P : et le chien a déclaré la maladie. Quand il a déclaré la maladie dans la commune, c’était dans un village de dioula. Le chien, immédiatement, il a contaminé les autres chiens qui sont dans le village. Donc, tous ces chiens-là ont commencé à faire la maladie. Et ils fonçaient vers les hommes, vers les animaux. Vous voyez mon inquiétude ? C’est pourquoi…

E : donc c’est la façon donc la propagation est très…

P : est très large. C’est-à-dire la propagation peut aller de façon spectaculaire, de façon, qu’un chien dans un village peut contaminer tous les chiens. Un chien, un autre chien, par ce qu’il ne reste pas dans une famille, il prend la route il va dans un autre village.

E : ok, ça c’est d’une façon générale, dans la communauté. Mais spécifiquement vous, qu’est-ce qui vous inquiète le plus, par rapport à la rage ?

**In Document:**
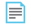
 18 KA_GD_HU_2409_T

Maintenant pour éviter la morsure du chien, est ce que pour éviter la morsure du chien, est ce que c’est facile ou ce n’est pas facile ou c’est plus ou moins facile ? On se protège pour qu’on ne soit pas mordu par le chien ?

P : Hééé, ça c’est vraiment difficile, c’est difficile.

E : C’est difficile?

P : Parce que si toi tu n’as pas de chien ceux qui sont dans la ville ont les chiens.

P : Ce que je peux dire, par exemple un jour nous sommes resté au centre, quand une fille quittait puiser de l’eau au marigot un chien est venu par derrière elle et la mordre, est ce qu’elle pouvait éviter cet accident ? Non parce que le chien lui a mordu par surprise, si tu veux interdire ça il faut que tu tues tous les chiens de ce contré.

E : Ok. Qui encore? P10.

P10: Si toi tu n’as pas de chiens, toux, ils sont dans le village ou tu peux partir chez quelqu’un qui a un chien, certains chiens toute personne qu’ils voient il faut qu’ils l’ambrassent, en cas où si tu vas trouver il n’ya personnes là-bas le chien peut te mordre.

E : P7 ?

P7 : Presque tout est dit, pour les chiens qu’on parle, certains chiens on peut les éviter, d’autres on ne peut pas comme ya une piqure pour les chiens pour la maladie qu’on appelle la rage, s’ils peuvent nous aider, s’ils ont les médicaments de piquer les chiens cela peut nous aider.

**In Document:**
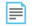
 56 KA_GD_MA1_2609_T

celui qui parvient à bien encadrer son chien comme les citadins le font. Nous, c’est pour la chasse que nous élevons les chiens. Dans les conditions normales, le chien doit être entravé pour n’être relâché que la nuit pour celui qui par exemple vit dans une cour pour la sécurité. Pour l’entraver encore le matin. Si on ne parvient pas à faire ainsi, ce comportement ne sera pas facile à réaliser.

**In Document:**
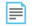
 45 KA_EN_AM_2409_T

A quel point êtes-vous préoccupés à contracter les maladies d’origines animales (bruit de personnes) ? Si ça vous préoccupe ou… ?

P : Moi ça me préoccupe hein ! Parce que moi j’évite, j’évite beaucoup les chiens surtout à cause de la rase, j’évite ça et quand je doute d’un animal, je ne consomme pas la viande même si je ne connais pas la nature de la maladie.

**In Document:**
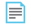
 29 KA_EN_VE_2409_T

E : parmi les deux formes de maladie zoonotique, quelle est la forme qui vous inquiète le plus ? Et pourquoi ?

P : par ce que c’est la rage

E : c’est la rage qui vous inquiète le plus ?

P : ça nous inquiète beaucoup.

E : pourquoi ?

P : par ce que, comme le système d’élevage fait que les gens n’arrivent pas à bien garder les animaux. Et quand un animal tombe malade, au lieu de rester à la maison, l’animal continue à mordre tout ce qui se trouve en route. Bétails, cailloux, quoi qu’il rencontre en route. Donc c’est très dangereux. Et c’est une maladie irréversible. Quand il se déclare, c’est fini, il est irréversible.

E : à quel moment de la maladie vous avez tendance à voir l’animal ?

**In Document:**
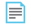
 42 KA_EN_LC_2509_T

P : Tu n’es pas chez toi ! Comment est-ce que dans une cour commune comme ça toi tu peux faire ton élevage ?

E : ça ne sera pas facile.

P : ça ne sera pas facile ! Même si tu as une chèvre comme ça là, tu vas l’abandonner dans la rue ? (avec insistance) Et puis ces animaux causent beaucoup de problèmes pour la circulation ici aussi. A chaque fois, ils font tomber des usagers, des motards. Des gens qui sont blessés et même des pertes en vies humaines.

E : Ok.

P : Haha ! Il faut trouver des lieux pour les gens, là où ils peuvent garder ses animaux.

E : Ok. Donc merci beaucoup pour le temps que vous nous avez consacré.

P : C’est moi qui vous remercie.

**In Document:**
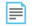
 48 KA_GD_FU_2509_T

E : quand on dit à quelqu’un tout à l’heure de ne pas manger la viande d’un animal malade, quand on dit à la population de Kankan de ne pas le faire, est-ce que ça sera difficile pour eux de le faire ou pas ?

Participantes : si c’est evia n’est pas difficile.

P10 : c’est notre méconnaissance qui nous fatigue. Parce que nous ne savons pas. Tu iras trouver la viande à la boucherie, tu vas l’acheter. Maintenant, comment toi tu peux savoir evia ne connais pas la viande pour dire ça c’est sain et evia n’est pas sain. Sinon la maladie du bœuf, quand tu achètes la viande, quand tu mets dans l’eau, c’est eviantt de la viande saine. La chair d’un bœuf malade, quand tu la laves elle eviant gluante. Donc tout le monde ne peut pas savoir cela. C’est ça. La viande saine, quand tu plonges dans l’eau, tu verras du sang rouge dans l’eau ; tu ne verras rien d’autre.

**In Document:**
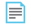
 20 KA_GD_FR_2509_T

P7 : Autre c’est la toux, certains animaux toussent, tu n’as pas le contrôle sur eux et tu dois les traiter comme tu te traite toi-même, des fois ils peuvent leurs bouches dans les bols, si ce sont les bols à manger et que toi tu mets la nourriture de dedans tu peux la contracter.

**In Document:**
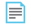
 29 KA_EN_VE_2409_T

E : ok, dans votre travail de vétérinaire, dans quelle mesures voyez-vous les maladies qui peuvent passer de l’animal à l’homme ?

P : oui. Dans mon métier de vétérinaire je remarque que, y a même des vétérinaires qui peuvent se contaminer. C’est-à-dire ils peuvent se contamine à partir des animaux malades et c’est au cours de l’examination de l’animal, quand un éleveur peut envoyer un animal, tel que le cas de la rage muette. La ratte muette, cette forme est dangereuse et contamine facilement le vétérinaire. Vous voyez. Quand il envoie son chien à la clinique pour nous dire : ah, mon chien est malade, je ne sais pas s’il a avalé un os, je ne sais pas si quelque chose l’a piqué. Donc, le vétérinaire si tu n’as pas beaucoup, tu n’es pas beaucoup informé, tu vas essayer de manipuler le chien et tu risques d’être contaminé.

**In Document:**
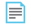
 29 KA_EN_VE_2409_T

P : je suis en contact avec des animaux malades à tout moment. Par ce que c’est des animaux de compagnie, même le chat. Le chat fait la maladie. C’est encore sa forme qui est plus dangereuse, par ce que, quand il fait la maladie, quand il fait la rage, il se cache et il est sur les lieux obscure et qui, généralement, arrive à mordre les enfants, c’est très fréquent. Par ce que ce sont les enfants ont l’habitude de voir un chat et avec leur bâton, ils peuvent le chasser.

E : ok, parmi toutes ces maladies zoonotiques, quelle maladie vous souci le plus ?

**In Document:**
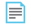
 56 KA_GD_MA1_2609_T

puisse que vous soyez responsable de famille, à votre avis qu’est-ce que les gens font pour se préserver des maladies ?

P1 : on peut aussi se protéger des maladies provenant des animaux en évitant toute viande dont la consommation n’est pas autorisée par un vétérinaire. Mais acheter une viande à la vente comme ça là, peut-être source des maladies inconnues. C’est ce que j’avais à dire

**In Document:**
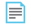
 56 KA_GD_MA1_2609_T

E : un autre comportement « nettoyer, voire désinfecter les couteaux ainsi que les surfaces ou on manipule la viande » est facile à réaliser ?

P2 : P2

E : que dit P2 ?

P2 : c’est n’est pas facile à réaliser parce que les femmes n’ont pas la même hygiène dans la cuisine tout comme tout le monde n’a pas la même hygiène lors du dépeçage. Les gens égorgent les animaux sans nettoyer le couteau avant de le mettre au fourreau tout comme beaucoup de femmes aussi peuvent mettre jusqu’à 3 jours sans laver leurs couteaux. Ce sont les conséquences de la négligence et du manque de contrôle.

**In Document:**
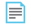
 20 KA_GD_FR_2509_T

PS : C’est utile.

E : Quelle est son utilité ?

P4 : Si on arrive à le faire c’est utile, ça peut nous prévenir de la maladie, ça peut nous prévenir de beaucoup de choses, parce que si c’est toi-même qui travail avec, mais si quelqu’un prête le couteau il se blesse avec, si le sang touche le couteau il va te contaminer la maladie.

E: Ok qui dit que ce n’est pas utile?

**In Document:**
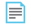
 37 KA_EN_LC3_2409_T.docx

P : je ne suis pas informé, parce que je suis parent de paysan aussi c’est une source de revenu pour nos parents le petit bétail ruminant comme les jusqu’au vache et autre là donc c’est une ressource pour la famille mais malheureusement.

**In Document:**
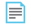
 12 KA_GD_HR_2509_T

: Si on prend le même comportement, la pratique de ce comportement est-il très utile, plus moins utile ou pas du tout utile ?

P7 : c’est utile car si tu nettoie l’enclos des animaux ça va diminuer tes dépenses et il permet d’augmenter la productivité animalière et souvent à l’approche des fêtes de tabaski si tu as beaucoup de mouton tu peux les vendre.

**In Document:**
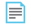
 1 KA_GD_MA2_2609_T.docx

E : pourquoi ?

P7 : ils diront que tu veux gaspiller la viande de leur bœuf. Ça c’est vraiment difficile. Parce que ce nous voyons, c’est que nous perdons le bœuf, et ça c’est difficile.

P3 : chez nous ici, la viande ne se trouve pas. Quand tu manges la viande cette année, il faudra attendre encore l’année prochaine. Quand ton bœuf meurt, toi tu jettes sa viande, hey (rires).

**In Document:**
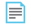
 20 KA_GD_FR_2509_T

E : Ok comment c’est facile?

P9 : Si tu les vaccines à tout moment ils auront longue vie, ils vont se reproduire tu va avoir des intérêts.

E : Mais est ce que c’est facile à réaliser pour tout le monde?

**In Document:**
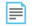
 48 KA_GD_FU_2509_T

P3 : ce n’est pas difficile. Assainir l’enclos des bétails, ce n’est pas difficile. Parce que quand tu assainis, tu assainis pour toi-même, du fait des maladies. Quand tu assainis, le problème de “diatakoly” et de moustiques dont on parle ne t’atteindront pas. Mais si l’endroit reste sale, ça va sentir, les moustiques vont te fatiguer, les “djatakoly” vont pénétrer chez toi. L’assainissement est une chose qui nous convient.

**In Document:**
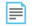
 48 KA_GD_FU_2509_T

P5 : assainir l’enclos des bétails, ce n’est pas difficile. Si tu veux tirer profit de ton bétail, il faut que tu t’en occupes, que tu assainisses le lieu. Quand tu assainis, toi-même tu profiteras de la bonne santé. Même quelqu’un d’autre qui trouve l’endroit propre aimera que l’enclos soit pour lui. Cela, c’est à cause de la propreté. Donc il faut assainir.

P7 : comme ce qu’elle dit, si tu assainis l’enclos du bétail, ensuite tu laves le corps de l’animal, puisqu’il ne suffit pas d’assainir le lieu seulement, si tu assainis son corps ; puisque l’animal pisse et chie là-bas, si tu assainis cet endroit et puis tu nettoie le corps même de l’animal, non seulement l’animal aura la santé. Puisque si tu vois que l’animal contamine l’être humain, il faut que l’animal même soit d’abord en bonne santé. Tu ranges d’abord là où l’animal dort, tu nettoie l’animal. Donc si ces deux sont propres, l’animal est bien entretenu, toi qui mange ça tu auras la santé. Mais si cet endroit n’est pas propre, toi-même être humain tu n’auras de santé.

**In Document:**
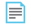
 1 KA_GD_MA2_2609_T.docx

P3 : la maladie de l’animal va se transmettre à l’homme.

P9 : euh ça c’est difficile. Ce n’est pas lié à la viande. Nous gagnons la viande. Ce qu’il faut dire, c’est le problème que nous avons. Sinon d’ici jusqu’à Kankan, ça fait 9 kilomètres. Tout le monde sait où se vend la viande. Tu peux aller à la boucherie et acheter la viande, surtout que nous avons des motos ici. C’est la pauvreté qui fait que nous ne jetons pas la viande. Quand toi tu envoies cette viande malade au marché, les gens vont acheter. Ils ne sauront pas ce qui a tué l’animal. Toi qui dépèces l’animal, dans ton travail, tu coupes la partie du foie qui est malade et tu jettes, dès que tu viens au marché et accroche ta viande, quelqu’un viendra te dire vend moi deux kilogrammes. En fait ceux qui achètent de la viande saine, toi tu n’as pas les moyens là. Tu n’as pas les moyens d’acheter la viande saine. C’est ce qui rend difficile. Sinon ce n’est pas difficile de jeter la viande d’un animal malade. Mais puisque tu n’as pas les moyens d’acheter la viande saine, tu te retrouves dans la situation d’acheter la viande malsaine. C’est ça.

**In Document:**
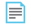
 18 KA_GD_HU_2409_T

Si nos animaux meurent quelque soit l’animal, creuser un trou et l’enterrer dedans est ce que le faire est difficile pour nous ou facile ?

P : Son cadavre ?

E: Enterrer son cadavre, est ce que c’est facile ?

P7: P7 Ce n’est pas facile pour nous ici, depuis moi je n’ai pas vu quelqu’un enterré son animal parce qu’il est mort malade.

P10: Le charbon a pris notre vache quand elle est morte on a creusé un trou et on l’a enterré, à part les cas de charbon on le fait pas.

Ps: Les gens prennent, la nuit les gens prennent. Brouhaha.

E : La nuit ?

P: La nuit les gens prennent, certains quand tu le jette la nuit d’ici le matin tu ne le verras plus.

**In Document:**
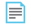
 1 KA_GD_MA2_2609_T.docx

P3 : Ils te chassent. Quand ils trouvent le berger, ils le chassent et prennent ses bœufs. Lorsque ces voleurs sont arrêtés, il n’y a pas de peines dissuasives. Tu peux arrêter un voleur aujourd’hui, un voleur de bœuf, tu l’envoies chez les chefs, quand il paie l’argent ils le libèrent. Donc cela fatigue énormément les bergers. Si les chefs arrivent à mettre fin à ce vol, ce sera bien. C’est pourquoi nous ne pouvons pas éloigner nos bœufs, ils doivent rester dans nos concessions.

**In Document:**
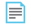
 5 KA_EN_LC2_2409_T

E : Certains de ces maladies, à votre avis sont-elles plus préoccupantes que d’autres ?

P : Ce qui doit vraiment préoccuper, c’est la maladie des bœufs : le charbon. Car les éleveurs, pour ne perdre, ne déclarent pas ces cas. Ils tentent de le vendre. C’est préoccupant, car ces cas peuvent facilement se rependre et provoquer une hécatombe.

E : Quand cette maladie frappe un homme qu’est ce qui peut t’arriver ?

P : Dans ce cas, si tu n’as pas un médecin compétant pour face à ta maladie. En demandant par exemple l’aliment qui a été à la base de ta maladie. Si tu restes à la maison à prendre les décoctions sans diagnostiquer la maladie, ça la situation sera ingérable.

**In Document:**
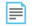
 48 KA_GD_FU_2509_T

E : toi tu ne dis rien depuis tout ce temps. Ou bien tu nous as oubliés ? Quand ton animal tombe malade, ou ta vaches tombe malade, qu’est-ce que tu fais ?

P5 : quand je l’égorge… Quand ma chèvre tombe malade, quand je l’égorge, tout le contenu abdominal, j’enlève tout ça. La chair, je l’assainis complètement ensuite je le prépare bien.

**In Document:**
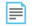
 56 KA_GD_MA1_2609_T

E : qui parlait ?

P6 : moi je veux parler de la maladie des poules qui les tue ou pour laquelle on les tue. Sans savoir les causes qui sont à la base de l’abattage des poules, les vendeurs de poulets les proposent, néanmoins à la consommation. On consomme de ces viandes de poules, sans savoir.

**In Document:**
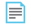
 9 KA_GD_MA_2409_T

P8 : il ya la vérité à ce niveau, beaucoup de gens faire l’élevage actuellement beaucoup ont cessé à cause des voleurs, toi tu vas élevé et que tu prends soin petit à petit un animal un beau jour quelqu’un vient volé çà et te déçois énormément, il ya vraiment beaucoup qui veulent faire l’élevage de vache ; mouton et chèvre mais quand tu penses au vole tu es découragé si non beaucoup de gens veulent faire cette activité, mais les voleurs nous ont fatigué féfé , tu peux voir dans un troupeau ils peuvent voler jusqu’à trente vaches donc tous çà décourager les gens à élever les animaux, donc les voleurs nous ont peur.

**In Document:**
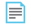
 1 KA_GD_MA2_2609_T.docx

Le premier comportement, c’est ce que vous voyez sur l’image ici. Séparer l’enclos des animaux de la maison des humains. Si on nous demande de réaliser cela, est-ce que la réalisation nous est difficile, pas difficile, ou bien c’est un peu difficile ?

P6 : séparer les animaux de notre maison, c’est très difficile. Nous partions derrière les bœufs au pâturage, de cet endroit jusqu’à la ville c’est trois kilomètres. Il n’y avait pas de vol de bétails. Mais de nos jours, garder les animaux dans la cour, mieux que ça garder le bœuf dans ta porte, ils viendront prendre ton bœuf et l’envoyer. Donc éloigner l’animal de nous en ce moment c’est difficile. Ça se faisait hier mais aujourd’hui ce n’est plus possible. Nous avons réellement peur du vol.

**In Document:**
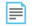
 18 KA_GD_HU_2409_T

Ceux qui ont dit que c’est difficile, lever les mains.

P: C’est des bœufs qu’on doit parler, tu sais l’homme avec par exemple le mouton, la chèvre tu ne peux pas t’éloigner d’eux ou t’éloigner d’eux d’une certaine façon même la vache là.

P : La vache et le porc, le porc lui, il sort se promener, il est bon hors du village à cause de son odeur, la vache aussi mais pas trop loin.

P : La vache moi je prends l’exemple sur nous, nous on avait les bœufs ils étaient éloigné de nous, les moutons aussi presque chaque année, chaque six mois, chaque trois moi on volait nos bœufs, parce que tu ne peux pas dire chaque nuit il faut que tu sortes pour aller voir les bœufs, notre père a ramené les bœufs à domicile, cela nous aider même si quelqu’un venait même à 2 heures du matin tu peux sortir pour les regarder donc ce facteur….Toux.

**In Document:**
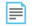
 12 KA_GD_HR_2509_T

E : oui P4

P4 : moi je dis cela est plus ou moins facile, car lorsqu’on éloigne les animaux de notre habitation nous avons peur

E : peur pourquoi ?

P4 : la peur que nous avons, ce le risque de se faire voler. Sans cela, cette pratique coute peu et est-elle hygiénique, dans la mesure où elle nous met à l’abri de l’odeur des urines et des excréments des animaux. A cause de la peur de se faire vole c’est pas facile sinon c’est facile.

E : ahan vous avez entendu P4

P10 : ce qu’il a dit est une belle vérité, moi je suis obligé de dormir avec mes veaux par peur de les voir volés. Donc, pour moi, cette pratique n’est pas facile à observer.

**In Document:**
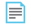
 42 KA_EN_LC_2509_T

P : Les vétérinaires. Ce sont les vétérinaires.

E : Ok.

P : C’est eux qui se chargent de non seulement de leur santé et…Voilà ! Quand il y’a problème, ils sont aussi concernés. Par exemple, ce que je vous ai expliqué toute suite, quand il y’a perte…

E : Vol de bétails.

P : Vol de bétails, tant tant tant ! Quand ils gèrent ça ici, ils sont appelés quoi, voilà.

6. KA2 **In Document:**
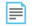
 48 KA_GD_FU_2509_T

P10 : tout ce qu’elles ont dit, c’est la vérité. Le vol que subissent les propriétaires de bétails ici, c’est beaucoup. Moi qui suis là, l’enclos est éloigné de notre maison. Mais je parle entre Dieu et moi, quand les voleurs viennent chercher mes poulets ou mes canards, ils peuvent en prendre jusqu’à quatre ou cinq en même temps. L’enclos est loin de notre habitation. Ensuite, les gens ne peuvent pas rapprocher les enclos des maisons si tu disposes d’un espace suffisant. Il y a certains endroits, c’est spacieux, il y en a d’autres qui ne disposent pas d’un grand espace. Donc, tu es obligé de rapprocher le bétail. Ce sont les deux points que je voulais ressortir.

P5 : ce sont les voleurs de bétails qui font peur aux éleveurs. Sinon, nous-mêmes on avait notre enclos au fond de notre concession. Mais à chaque fois les voleurs de poulets cassent la porte de l’enclos. Ils ne volent à aucun moment si ce n’est quand les poulets commencent à grandir, au moment où tu dois profiter d’eux ; c’est en ce moment ils viennent les prendre.

**In Document:**
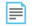
 56 KA_GD_MA1_2609_T

P : ce qui fait que c’est difficile c’est l’envie de manger de la viande qui n’est jamais satisfait.

P6 : ce n’est pas facile. Ce qui fait que ce n’est pas facile. Nos besoins dépendent essentiellement de la chasse de ces animaux. Si vous faites un tour chez moi vous y trouverez une perdrix et j’ai déjà empoché son prix. Nous demander de renoncer à ça ne marchera pas c’est ce que j’avais à dire.

P2 : ce n’est pas facile car c’est notre source de revenus. Toute la pluie de la nuit passé m’a battu, quand vous faites un tour chez moi tout de suite vous trouverez un lapin. Quand on m’achètera cela, ça me permettra d’avoir 5litres à la station-service.

**In Document:**
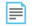
 48 KA_GD_FU_2509_T

on dit « d’écarter l’enclos des animaux de l’endroit où nous passons la nuit ». Vous les femmes qui êtes là, si on vous demandait si réaliser ce comportement à Kankan était difficile à faire, facile à faire ou un peu difficile à faire.

P7 : ce que tu dis là, c’est difficile à réaliser. Pourquoi c’est difficile à faire, nous les propriétaires de bétails travaillons jour et nuit. Chaque année je fais la vaccination du bétail dans tout kankan. Tu vas aller chez certaines personnes, tu entres d’abord dans la maison, tu travers pour passer par derrière et te rendre à l’enclos. J’ai demandé à la femme et disant : ah maman, pourquoi fais-tu ça ? Chaque fois qu’on arrive, il faut qu’on traverse ta case pour arriver à l’enclos. Tu passes la nuit avec tes animaux. Elle a répondu qu’elle a peur des voleurs. Que les voleurs l’ont trop fatigué. Qu’à chaque fois ils viennent voler ses moutons. Que c’est pour cela qu’elle aussi a pris tout son bétail et les a rapproché. C’est pour que dès qu’un voleur arrive, les moutons vont faire du bruit ou les chèvres vont faire du bruit. Avec ça, elle peut se rendre compte que les voleurs sont arrivés. Je lui ai dit alors il faut éloigner l’enclos de ta couchette. Sinon le caca et l’urine que ces animaux déversent, beaucoup de choses te parviendront : les moustiques viendront, les insectes piquants vont venir, ils vont rentrer dans ta maison et te transmettre la maladie. Elle a répondu qu’elle préfère cela au vol de son bétail, moutons et chèvres. C’est pour cela il lui est difficile d’éloigner l’enclos de l’endroit où elle dort. Donc il y a beaucoup de cas, les enclos sont à la rentrée de la maison.

**In Document:**
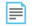
 56 KA_GD_MA1_2609_T

aujourd’hui, certains souhaitent même avoir l’habitation de leur bétail devant leurs portes. Nous savons que c’est nuisible à nous. Mais cela ne compte pas pour nous. Ce qui compte ce sont nos bétails. Ils ne préoccupent plus que notre santé. Par ce que vouloir avoir aujourd’hui son bétail dans un pâturage en dehors du village, le jour que tu découvriras une disparition, cela ne t’étonnera pas. C’est s’il n’y a pas de disparition qui va t’étonner. Voilà pourquoi nous avons rapproché nous animaux. Avant les gens se mettaient ensemble pour avoir un pâturage commun hors du village et chargé un peulh de la surveillance. En ce moment les camaras n’étaient pas nombreux, mais aujourd’hui eux ils ne pardonnent pas les pratiques comme ça là

**In Document:**
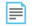
 20 KA_GD_FR_2509_T

Ya une différence entre eux.

E : Oui ya quelle différence?

P3 : Si tu dis de les éloigner trop de nous ça ne se fait pas du tout, si tu les éloignes certains moments vont arrivés, parce que nous, nous avons éloigné nos chèvres et nos bœufs de nous, ils ont volé nos chèvres .

E : Donc toi tu veux que dire que c’est utile?

P3 : C’est utile.

**In Document:**
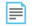
 43 CN_GD_FU_2009_T

E : ok. On va passer. Maintenant, l’’autre image, vous savez dans certains endroits, on fait bouillir le lait pendant 30 minutes avant de le boire pour ne pas que ça transmette la maladie. Maintenant, est-ce que quand on demande aux gens aujourd’hui à Conakry, de toujours faire bouillir le lait pendant 30 minutes avant de le boire, cela sera facile à réaliser ?

P4 : ce n’est pas facile, pourquoi ? Parce qu’à quel moment cela se fait ? Quand on extrait le lait, avant qu’il ne se fermente, avant que ça ne soit du lait caillé, c’est en ce moment qu’il faut le faire bouillir pendant 30 minutes. Mais avant que ça n’arrive à Conakry ici, ça trouve que c’est déjà caillé donc on ne peut plus le faire bouillir.

**In Document:**
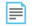
 10 CN_GD_HU_1909_T

P2 : l’hygiène. Ce que nous, nous faisons dans notre quartier, nous veillons à l’hygiène, qu’il y ait ans le quartier. Quand il y a l’hygiène dans le quartier, il y a certaines maladies dont on peut se préserver. Aussi, tous les chiens qui errent au milieu de nous, s’ils ne sont pas vacciner, qu’on les tue et qu’on les enlève au milieu de nous.

E : oui qui d’autres ?

**In Document:**
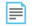
 60 CN_GD_HU1_1909_T

P 2 : Si tu as un animal, si tu n’as pas d’enclos, il faut au moins disposer d’un endroit où il faut l’abriter. Et le propriétaire est obligé de rendre propre cet endroit. Ceux qui n’on pas d’enclos sont obliger de vivre avec les animaux, et quand on vit avec un animal, on est obligé d’y maintenir l’hygiène.

P4 : Il s’agit de désinfecter l’enclos. Alors qu’on a dit ici qu’il n’est pas facile de se faire un enclos ici. Nous vivons avec les animaux. Et ce qui désinfecte peut constituer un poison pour nous. Ce que l’animal peut supporter en terme de santé, l’homme ne peut pas supporter. Et le désinfectant utilisé, s’il peut être supporté par l’animal, l’homme ne peut pas le supporter. C’est impossible

**In Document:**
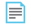
 43 CN_GD_FU_2009_T

P4 : c’est ce que je dis comme ça, ce n’est pas facile, parce que toi tu ne sais à quelle heure le propriétaire du chien libère son chien, et tu ne peux pas savoir à quel moment vous pouvez vous rencontrer. Donc, tu ne peux pas prendre des précautions par rapport à ça, parce que tu ne sais pas à quel moment il peut venir, tu ne connais pas à quel moment le propriétaire le libère. Si c’est un chien qui est dans la rue, qui n’est pas domestique, si tu vois ça quand même, tu peux l’éviter en marchant à son côté opposé. Mais ceux qui sont avec les gens, quand les propriétaires les lâchent les matins par exemple, toi tu ne sais pas qu’ils sont sortis, donc si vous vous rencontrez et qu’il te morde, là ce n’est pas de ta faute.

**In Document:**
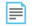
 43 CN_GD_FU_2009_T

E : ok. Une autre image, manger de la viande d’animaux malades. Quand on dit aux gens à Conakry d’éviter de faire ça, est-ce que cela peut être facile ?

P5 : si je comprends bien votre message, c’est d’arrêter de manger la viande d’animaux déjà malades ?

E : oui

P5 : on ne peut pas arrêter de manger ça, parce que dans nos abattoirs à Conakry ici, ils ne prennent l’initiative d’égorger un animal que quand il tombe malade. Donc quand ils égorgent, c’est la viande là qu’ils mettent pour nous sur le marché. Et c’est cette maladie que nous, nous achetons maintenant pour venir cuisiner et manger. Mais si l’animal est chez toi, quand il tombe malade, tu peux lui donner les médicaments ou quand il meurt de lui-même, tu le jettes. Mais dans ville de Conakry, même si le bovin meurt de lui-même, ils n’osent pas le jeter parce que c’est leurs millions qui s’envolent, il faut qu’ils ne la vendent.

P2 : je prends un exemple sur le bovin. Il y a certains bovins qu’on tue ici alors qu’ils sont en gestation. Pourtant, normalement le bovin, quand il est en gestation, tu n’as pas le droit de l’égorger, parce qu’on voit tout ici. Déjà, même là où on abat les bovins, à l’abattoir, même les mesures à prendre pour se préserver des maladies, ils ne les respectent pas du tout, puis que là où toutes les toilettes sont drainées, ce que nous appelons la mer, quand ils finissent d’égorger les bovins, c’est là-bas qu’ils vont laver leur couteau. Le même couteau est utilisé après pour égorger l’animal, le même couteau est utilisé pour découper la viande. Si toi tu manges ça, ça va te transmettre ou pas la maladie

**In Document:**
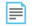
 30 CN_GD_MA3_2109_T

P4 : sur ce plan, concernant cette vérification, ce sont les éleveurs qui réalisent le plus souvent. Par ce que nous une vache et un mouton ne durent pas entre nos mains. Il peut débarquer aujourd’hui et être vendu aujourd’hui. Ça peut être deux jours, trois jours, 5 jours ou une semaine. En tout cas ça ne dure pas entre avec nous. Cette vérification, cette vaccination avant même que l’animal ne tombe malade revient aux éleveurs, nous sommes des vendeurs, quand ça vient on écoule. C’est pourquoi chaque samedi nous partons dans les marchés hebdomadaires, nous débarquons le Lundi et d’ici le Samedi, le plus souvent on aura écoulé tout. Ce cas, on ne dit pas que ça ne nous importent pas mais ce n’est pas trop.

**In Document:**
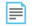
 10 CN_GD_HU_1909_T

Pour la première image ou premier comportement, il s’agit d’éviter de manger la viande d’animaux malades. A votre avis, selon vos connaissances sur ce qui se passe dans votre communauté ici, est-ce que là c’est facile ou pas à réaliser ou plus ou moins facile ?

P2 : ce n’est pas facile à réaliser. Comment ? Parce que toi tu ne vois que la viande, l’animal n’a pas été tué en ta présence, tu ne sais pas si pendant qu’on le tuait il était malade ou pas, tu ne vois que de la viande à acheter, tu ne sais pas si ça a été inspecté ou si le vétérinaire avait contrôlé ou pas…

**In Document:**
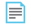
 30 CN_GD_MA3_2109_T

E : les animaux de brousse figurant sur cette image. Est-ce facile d’éviter de manger la viande de ces animaux ?

P6 : moi je pense qu’éviter de manger cette viande est facile, par ce que tu vois chaque viande que tu veux acheter et ne te vend pas seulement, dans la plus part des cas il y a une différence. On te dit que ça c’est de la viande de bœufs, de chèvre… et c’est aussi de la viande de brousse on te le dit, on précise. Chacun vient avec son besoin et à mon avis c’est très facile.

P1 : pour moi c’est facile, par ce que ces animaux de brousse mentionnés sur cette image, si ce n’est pas quelqu’un qui le veut… la plus part des musulmans ne mangent pas cette viande. Et là où on vend, il y a une séparation, même si c’est dans le même marché, on met toujours cette différence.

P2 : c’est facile. Pourquoi c’est facile ? Partout on vend cette viande, que ça soit au bord de la rue, sur les tables dans les marchés, on te dit de quelle viande il s’agit : du lapin, de la gazelle et si c’est du singe on te dit tout. Et c’est toi maintenant qui sait si tu vas acheter ou pas. Dans ce cas c’est facile.

P3 : éviter de manger la viande de ces animaux, c’est facile.

**In Document:**
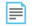
 43 CN_GD_FU_2009_T

P4 : Les gens envoient et les consommateurs ne savent pas généralement que c’est de la viande de brousse. Moi par exemple, quand j’ai envie de manger de la viande, j’achète sans demander c’est quelle viande en gardant à l’esprit que c’est quand même de la viande. Provenant d’un animal malade ou pas, je ne peux le savoir. C’est donc difficile à réaliser

**In Document:**
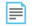
 61 CN_GD_FU1_2009_T

P5 : Exemple, comme les vaches que nous tuons. Souvent certaines vaches non vaccinées sont abattues et leurs chairs vendues. En plus ces foies importés, nous ne savons pas si les animaux à l’origine étaient malades. Ca peut donner la cirrhose de foie. Il ya de ces vaches qui ont leurs foies tout rouge et d’autres tout noir. Nous recevons beaucoup de qualités chez nous ici. Nous nous ne pouvons pas avoir ce qui est bon ou n’est pas bon. C’est pourquoi il y’a actuellement beaucoup de maladies chez nous ici car nous consommons ces foies importés sans savoir d’où ça vient ; Est-ce que ca été vaccinés à l’origine, nous ne savons rien.les maladies diabète et cirrhose sont très répandues actuellement. Quand tu les consommes souvent tu as de haut-le-cœur, moi c’est à cela je pense

**In Document:**
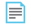
 60 CN_GD_HU1_1909_T

Plus ou moins difficile parce qu’il n’ya pas de brousse ici certes mais si j’achète de la viande avec un marchand ambulant, je ne peux men rendre compte de l’origine de la viande

P1 : Plus ou moins difficile. Si je rends visite à quelqu’un et qu’il me tende de la bonne viande je ne peux vérifier son origine

**In Document:**
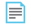
 31 CN_GD_MA2_2109_T

E : ok. Maintenant, à supposer que nous savons, on nous informe, éviter de manger cette viande provenant des animaux de brousse est utile pour notre santé ?

P2 : rester sans manger est très avantageux pour nous. Mais si tu ne sais pas, tu as demandé de la viande mais tu ne connais ce qu’on a égorgé. Si de la viande de brousse, toi tu ne sais pas, c’est alors facile et difficile pour toi.

E : nous disons est-ce que c’est utile si nous ne mangeons pas la viande de ces animaux ?

**In Document:**
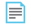
 53 CN_EN_LC3_1909_T

E : Qu’est ce qui empêcherai les populations de suivre les directives des autorités

P : les gens peuvent refuser de respecter ces directive si par exemple tu sais comment soigner les poules a traves le citrons et l’eau de canarie tu vas d’abord essai de soigner tes volailles par ce que abattre comme ça les animaux ce ne pas bon, mais si tu essaie et que ça ne marche pas tu peux te soumettre à la décision des autorités

E : Dans quelle mesure feront –il confiance à ce que disent les autorités ?

P : les gens qui ne savent pas qu’on peut soigner le poulet à travers les méthodes que je viens de citer peuvent croire directement à ce que disent les autorités et abattrons les animaux, mais celui sait comment les soigner tu utiliseras d’abord ces méthodes pour éviter d’abattre les poulets

**In Document:**
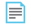
 10 CN_GD_HU_1909_T

P5 : pour moi, ce n’est pas facile, parce que quelqu’un peut tuer un animal malade, et toi qui viens acheter, tu ne sais pas. Tu achètes, tu envoies à la maison, tu la prépares et si c’est mal cuit, si tu la manges, tu contractes la maladie, ou même si tu prépares bien, mais s’il l’animal a été malade, tu contractes la maladie aussi. Donc vraiment, ce n’est pas facile à réaliser du tout, par qu’on n sait pas où l’animal a été tué ou s’il a été vacciné ou pas av les vétérinaires ; je me limite là.

**In Document:**
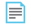
 10 CN_GD_HU_1909_T

P3 : je reviens toujours sur le ténia. Euh… apparemment, on constate que ceux qui vendent des bœufs, une fois qu’on sent que l’animal est malade, on n’appelle pas les vétérinaires, au contraire, on l’abat rapidement pour ne pas que l’animal meurt. Et une fois que l’animal est contaminé, qu’il n’a pas été consulté, nous les consommateurs, on va aussi au marché, on achète, et si ça trouve que la viande n’est pas bien préparée, toi aussi tu peux facilement contracter la maladie à travers de la viande mal préparée. Et surtout aussi les brochettes qu’on vend au milieu des femmes, on trouve le sang. Communément on dit que quand il y a le sang, ça augmente la vitamine, mais au contraire…, donc ça aussi, ça a vraiment un impact majeur pour la facturation de ces animaux.

**In Document:**
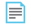
 60 CN_GD_HU1_1909_T

Pas facile du tout parce quand tu te rends dans un centre de santé, ils te disent d’aller dans un cabinet veterinaire et ceux-ci demandent à voir l’animal

**In Document:**
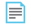
 60 CN_GD_HU1_1909_T

P 3 : Ce n’est pas facile parce qu’il n’y a pas de cliniques vétérinaire à proximité. Il n’y en a même pas

P 10 : Ils te demanderont de voir le chien qui a mordu d’abord avant les soins

P 4 : L’argent exigé aussi pour bénéficier de soins est exorbitant car il peut dépasser le prix d’un sac de riz

**In Document:**
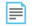
 60 CN_GD_HU1_1909_T

Ok P4, merci. P5 veut dire quelque chose

P5 : Comme l’a dit mes confrères, diffuser l’information au sein de la population pour éviter ces maladies, c’est utile. Mais ce qui est plus utile, il faut que les ONG unissent leur force à celle du gouvernement. On peut éviter la maladie la maladie mais difficile d’éviter un chien abandonné à lui-même. On n’a pas de moyens de les éviter. Ce n’est pas facile aussi pour les individus de prendre des initiatives pour faire vacciner les chiens errants. Ce qui pourrait le facilité, c’est la conjugaison des efforts. Comme la l’action de Mouctar Diallo. En plus, on peut faire du porte à porte pour recenser tous les propriétaires d’animaux afin de savoir ce qui dans le cadre et ce qui ne l’est pas. Comme ca, ce serait facile d’éviter. Mais tant que les chiens sont abandonnés et que les chats errent sur nos toits, ce ne serait pas facile.

P 9 : Pour ce qui est des animaux domestiques, il faut reconnaitre qu’il ya aujourd’hui beaucoup de vendeurs de poulets, et des poulets de tout genre. Parfois nous ignorons même leurs provenances. Alors que dans des pays comme le Burkina, leur importation a été minimisée. Là-bas, les vétérinaires sont très impliqués dans la gestion des animaux

Et pour les autres animaux, comme la vache par exemple, l’Etat doit aussi assurer un bon suivi des bêtes de leur provenance à l’abattoir. Car il ya de ces abattoir chez nous, à observer les conditions d’hygiène, on a plus envie de consommer de la viande. Valable pour les endroits où ces viandes sont vendues. Ailleurs les viandes sont vendues dans des vitres, mais ici c’est à l’air libre : l’air pollué de microbes et les mouches aussi en apportent et le soleil aussi

**In Document:**
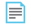
 3 CN_GD_MA_2009_T

Eviter de manger la viande d’animaux malades, c’est facile, plus ou moins facile ou pas facile à réaliser ?

P5 : c’est facile à réaliser.

E : comment ?

P5 : parce que si tu connais et que vois l’état de l’animal, tu vas éviter.

P6 : faire quoi ?

E : éviter de manger la viande d’animaux malade, c’est facile, plus ou moins facile ou pas facile à réaliser ?

P5 : si tu connais, c’est facile à réaliser.

E : quelqu’un a un avis contraire, pas facile réaliser ?

P : c’est facile à réaliser par que chez nous les vendeurs souvent les vétérinaires contrôlent les animaux avant la vente.

**In Document:**
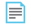
 53 CN_EN_LC3_1909_T

E : Quelles sont les contraintes auxquelles font face les gens de votre communauté en ce qui concerne la gestion des animaux

Dans la communauté il y a des gens qui ont des chiens qui ont des maladies et si ces chiens se frottent contre une personne, il vous transmet la maladie, il y a aussi des chiens abandonnés dans la rue quand ils te mordent ou quand ils mettent leur gueule dans la nourriture, la personne qui mangera cet aliment sera contaminer par la maladie et sa deviendra des problèmes

Connaissance et perception de risques

**In Document:**
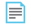
 3 CN_GD_MA_2009_T

E : Eviter des morsures de chien c’est facile à réaliser, pas facile à réaliser ou plus ou moins facile à réaliser ? éviter les morsures de chien comme on l’avait dit au début. Des maladies qui peuvent être transmises de l’animal a l’homme.

P4 : ce n’est pas facile. C’est seule Dieu qui peut te protéger.

P3 : ce n’est pas facile car tu peux rencontrer un chien errant dans la rue qui peut t’agresser. Donc sauf Dieu peu sauver.

P2 : ce n’est pas facile car tu peux rencontrer un chien errant dans la rue qui peut t’agresser. Certain quand vous vous rencontre et que tu cours, il va suivre. Quand tu fonce, il peut te mordre. Ou bien tu peux le trouver coucher sans le savoir quand tu le touches, il te mord. Donc ce n’est pas facile.

**In Document:**
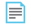
 10 CN_GD_HU_1909_T

E : Maintenant, pour la quatrième image, on nous dit d’éviter les morsures de chiens. Est-ce que selon vous ce comportement est facile à réaliser ?

P7 : pour moi, c’est facile à réaliser, parce que le chien, c’est chez toi qu’il se trouve, c’est pour toi t ta famille. Mais quand une autre personne doit venir chez toi, il te faut le protéger pour qu’il rentre chez toi, pour ne pas que le chien le morde. Deuxièmement, les chiens errants qui sont dans les rues, si tu ne les provoques pas, ils ne te provoquent pas ; donc c’est facile.

E : oui qui d’autres ?

P6 : ce n’est pas facile, parce qu’ici, c’est les chiens errants qui sont nombreux, et moi je sais en venant au boulot, combien de chiens je dépasse. Pourtant il y a certaines personnes, elles ont naturellement peur de chiens. Donc, tu peux voir un chien et tu as peur, et si tu prends peur, il peut t’offenser ; donc ce n’est pas facile

**In Document:**
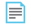
 49 CN_EN_LC2_1909_T

Moi tout ce qui est manger je n’achète pas à avaria, tout ce qui est avarie on ne doit pas manger, si tu gagnes une maladie tu as cherché. Nous en ce moment on n’était obligé de sensibiliser pour ne pas que les gens achètent ces poulets-là, c’est quelque chose déjà qui est rejeté par les Européens donc ce n’est pas des maladies immédiates, quand tu manger ce poulet là ça réagir sur toi, mais l’africain ne connait pas ça, il y a des gens qui disait que c’est faux et que si tu fais bouillir avec du piment que la maladie ne va pas t’attrapé. C’est comme ça que certains nous répondaient donc mais notre possibilité c’était de sensibiliser, d’informer les gens sur les dangers qui était autour de ça.

**In Document:**
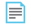
 33 CN_GD_MA1_2109_T.docx

P1 : c’est facile à réaliser parce qu’on ne te force pas à manger. C’est toi-même qui regarde quand tu parts acheter la viande ou acheter à manger. Tu regardes toi-même.

P : c’est facile à réaliser.

P : c’est plus ou moins facile parce que c’est dans ta famille que tu peux contrôler mais quand tu sors hors de chez toi, ce n’est pas facile.

**In Document:**
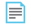
 30 CN_GD_MA3_2109_T

P3 : c’est comme ils l’ont dit, éviter est difficile, par ce que tu l’as pas égorgé, tu vois de la viande crue. Tu ne sais pas s’ils l’ont contrôlé ou vérifié. Il y a aussi le cas des poulets et foies importés dont on ne sait pas depuis combien d’années ils sont égorgés. Et si ceux à qui on a la confier la gestion de la santé de population concernant, particulièrement, la viande, cela va rendre la situation compliquée et difficile à éviter.

**In Document:**
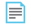
 33 CN_GD_MA1_2109_T.docx

P : excuse grand frère, tu peux voir quelque chose malade et manger ? tu ne peux pas j’espère ! nous avons dit qu’une personne peut éviter chez soi mais quand elle sort de chez elle, c’est pas facile. Parce que tu peux aussi acheter à manger.

**In Document:**
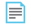
 31 CN_GD_MA2_2109_T

Nous allons parler d’un autre point. Qu’est-ce qui vous pousse à vous décider de faire un test de santé des animaux ? Si vous faites des tests.

P6 : ce qui fait ça, c’est pour éviter que les animaux ne soient malades.

E : est-ce qu’il vous arrive de faire un test pendant que l’animal est en bonne santé pour une vérification ?

P8 : nous faisons des tests pour éviter que nous vendions un animal malade pour ne pas que les acheteurs mangent ça sans le savoir et qu’il y ait une maladie contagieuse.

E : quelqu’un veut dire quelque chose ?

P4 : là où nous les achetons, ils nous donnent des papiers qui certifient que l’animal est en bonne état.

**In Document:**
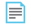
 30 CN_GD_MA3_2109_T

Parlons de nous et des autres. Pensez-vous que c’est facile d’éviter de manger de la viande des animaux malades ?

P1 : moi je dis que c’est facile à faire. Pour veiller sur ta vie et la sauver, moi je dirais que ce n’est pas difficile pour soit.

P6 : moi je dirais qu’éviter ça n’est pas facile, par ce que quand on envoie les animaux à égorger, toi tu n’es pas là. Tu ne connais pas l’état dans lequel ces animaux sont arrivés, est-ce qu’ils étaient malades ou pas avant de venir à l’abattoir, tu ne sais rien, tu ne vois que la viande. Ce qui peut aider la population dans cette situation, c’est le service des vétérinaires. Ils font leur contrôle avant et après avoir égorger la vache avant que la viande ne soit livré au marché, sinon nous nous allons en manger et… actuellement toutes les maladies viennent de la nourriture. C’est ce que j’avais à ajouter.

P4 : de ce côté, ce sont les vétérinaires qui peuvent nous aider.

E : c’est difficile d’éviter ou pas ?

P4 : ce n’est pas difficile, mais ce sont les vétérinaires qui peuvent t’aider. Si tu es informé, tu n’achètes pas. Mais si tu ne sais pas, ce sont les vétérinaires qui peuvent ces animaux on ne les mange pas, ces autres il faut les manger.

P2 : c’est difficile d’éviter, pourquoi, par ce que c’est la viande toi tu vois, tu ne vois quand on n’a pas égorgé. Tu ignores si l’animal était malade ou sain. Dans ce cas éviter n’est pas facile pour toi.

E : alors qui sont ceux qui pensent que c’est facile ?

**In Document:**
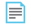
 17 CN_EN_LC1_1909_T

E : Ok quel rôle avez-vous joué, toujours nous demandons le rôle que vous avez joué dans la communauté pour le soutien des informations envoyées par les autorités comment les gens de votre communauté ont-ils réagit par les nouvelles des autorités?

P: Les gens ont réagi d’une façon positive par ce que l’information est la source nourricière quand on n’a pas une bonne information on tombe dans le trou, mais lorsqu’on n’a une bonne information on peut se sentir heureux donc l’information est parvenue du central nous avons relevé cette information au niveau de la communauté et chaque membre de la communauté a pris soin de nous écouter et suivre les recommandations données à cet effet.

**In Document:**
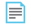
 58 CN_EN_AS_2109_T

Si vous apprenez une information tout de suite, comment vous, vous pouvez décider que cette information est fiable ou non ?

P : quand j’apprends une information tout de suite, j’essaye de procéder à des analyses, voir jusqu’à quel niveau-là, l’information est tenace, à mon tour j’essaye de voir aussi, de, de transmettre cette information parce que quand je sais que c’est fiable, je le transmets.

E : oui c’est ce que j’aimerais vraiment comprendre, si je comprends un peu, d’abord vous cherchez vous-même à vérifier l’information ?

P : oui

E : et comment vous le faites ?

P : je fais, je…, je vérifie cette information, toujours avec ma population ou la radio ou mes sup…, je, je, mes chefs hiérarchiques au centre de santé

**In Document:**
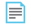
 43 CN_GD_FU_2009_T

P3 : pour moi ce n’est pas facile, parce qu’il y a certains qui sont plein de paresses, ils ne sont pas propres. Ils peuvent travailler avec le couteau tout à l’heure et le déposer comme ça, et après, quand ils en ont à nouveau besoin, ils le prennent et ils travaillent avec sans contrôler ; ils ne l’entretiennent pas.

P4 : normalement un couteau, c’est une seule personne qui doit travailler avec. Mais, en général ce n’est pas une seule personne qui travaille avec, parce que tu peux venir tout à l’heure, tu es en train de préparer, tu es pressée, mais tu ne vois pas ton couteau, il te faut donc aller chez ta voisine emprunter son couteau rapidement. Vous voyez ? Donc ce n’est pas facile.

**In Document:**
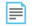
 60 CN_GD_HU1_1909_T

Le poulet, est très utile pour nous. Le phacochère, nous l’élevons, nous l’utilisons, il est aussi utile. La vache, nous l’elevons et nous l’utilisons notamment dans nos travaux champêtres. Donc ils sont utiles pour nous. La chèvre et le mouton, nous pouvons l’élever, le commercialiser et utiliser leur lait. Mais la viande de vache, quand on atteint un certain âge, il faut éviter de la consommer. Ce sont des viandes à consommer avec modération à cause de la quantité de graisse qu’elles contiennent au risque d’avoir des hémorroïdes.

**In Document:**
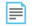
 10 CN_GD_HU_1909_T

E : laissons d’abord les moyens. Partons des réalités telles qu’elles sont et que vous la connaissez dans votre communauté…

P4 : si c’est comme ça, ce n’est pas facile à réaliser, parce qu’il y en a qui élèvent les animaux aujourd’hui alors qu’ils sont en location.

P2 : ce n’est pas facile à réaliser, par qu’il y a de ces animaux, nous sommes obligés de vivre avec eux, tels que certains animaux domestiques (comme on les appelle), donc, on est obligé de vivre ensemble. Maintenant s’il y a s choses ans ça qui sont mauvaises, c’est des dispositions qui doivent être prises pour ça. Mais ce n’est pas quand même qu’ils vivent à part t nous aussi à part.

P1 : Les animaux, nous cohabitons avec eux. Donc, tel que tu prends soin de toi, c’est ainsi que tu dois en prendre soin. Si tu fais bien son entretien, à certain niveau, si tu en as besoin, tu peux en faire bon usage. C’est quand tu les abandonnes que quelque chose de mauvais peut leur arriver. On ne doit pas s’en éloigner, on n’est tous ensemble.

**In Document:**
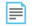
 10 CN_GD_HU_1909_T

E : Qu’en est-il des pratiques locales ? Comment traditionnellement, vous faites pour vous protéger de ces maladies qui viennent des animaux ?

P4 : Ce dont nous parlons, il s’agit du chien et du chat, ce sont des animaux qui vivent avec les hommes. Et nous le savons, ils sont autant utiles pour nous qu’ils sont dangereux ; mais nous connaissons l’importance du chien. Qu’est-ce que la population, un père de famille doit garder à l’esprit ? Seul celui qui peut avoir un contrôle sur un chien a le droit de l’élever. Seul celui qui peut avoir un contrôle sur un chat a le droit de l’élever. Il y a trop de chats abandonnés, pourtant, le chat est très utile dans la maison, puis qu’il aide à chasser les souris.

**In Document:**
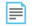
 60 CN_GD_HU1_1909_T

Qu’est ce qui peut motiver quelqu’un à faire appel à un vétérinaire ?

P 9 : Il ya beaucoup d’éleveurs qui aiment leur animal et donc ils ne négligent pas la maladie de leur bête

E : Et qu’est ce qui peut faire qu’ils ne font pas appel au vétérinaire ?

P 5 : Les motifs d’élevage ne sont pas les mêmes. Les uns élèvent un mouton par exemple pour en faire un sacrifice. Quand cet animal tombe malade, cet éleveur cherchera à le soigner.

D’autres élèvent pour leur consommation, donc quand ces animaux tombent malades, ils les tuent et consomment aussitôt.

D’autres c’est pour commercialiser, donc quand ca tombe malade, pour ne pas que ca soit une perte, il fait appel à un vétérinaire pour soigner l’animal

Mais si ce sont des animaux que l’on ne consomme pas, ils le chassent du domicile ou cherche à tuer pour aller jeter.

P1 : Je lui rejoins mais certains jettent pourquoi aussi, c’est à cause du manque d’information car si vous dites à certains ici que l’on peut envoyer un coq par exemple dans un certain de santé, ils vous traiteront de fou. En plus les gens ne peuvent pas acheter des petits poussins à environ 10 000 fg et quand ils tombent malades que les frais de soins s’élèvent à 50 000 fg. Ils n’accepteront pas cela.

E : Y’a-t-il autres raisons ?

P 3 : Si l’éleveur, élève dans l’objectif de vendre sa bête, il cherchera toujours a sauvegardé ses intérêts.

P 1 : Si c’est quelqu’un d’informé qui sait que l’on peut soigner un animal et qu’il a peur de Dieu, sachant quand offrant la viande d’un tel animal à d’autre ça peut donner de la maladie, la personne ferait soigner l’animal. Mais je n’ai pas vu de personne comme ca ici d’abord

**In Document:**
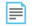
 10 CN_GD_HU_1909_T

E : ok. Maintenant, à quel moment les gens décident de tuer leur animal malade ? Dans quel genre de situation ?

P2 : Quand il n’y a plus d’espoir

P1 : Quand il n’y a plus de moyens. Au lieu qu’il ne s’affaiblisse de lui-même au point de ne plus avoir d’importance pour les gens, donc les gens préfèrent tuer pour le manger afin qu’il serve au moins à quelque chose.

P3 : Euh…, tuer l’animal alors que tu sais que vraiment il est malade, ça c’est dure…, surtout si c’est la vache, c’est qu’il n’y a plus d’espoir c’est vrai, mais pour l’argent, il y en a d’autre, pour l’argent, il sait que si l’animal meurt comme ça, il ne va rien bénéficier, alors il va anticiper, il tue l’animal et puis il revend la viande, voilà ! C’est ça aussi l’impact de la pauvreté.

**In Document:**
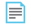
 33 CN_GD_MA1_2109_T.docx

couvrir les coupures ou les blessures sur la peau lorsque vous manipulez de la viande c’est facile, plus ou moins facile ou pas facile.

P : c’est facile

P2 : c’est facile parce que tu ne peux pas laisser le sang couler sur la viande. Donc tu dois chercher la coule pour coller la plait à l’hôpital.

E : donc nous sommes tous d’accord que c’est facile à réaliser.

Alors c’est facile à réaliser c’est utile, plus ou moins utile ou pas utile ?

P : c’est utile car tu te protèges et tu protèges tes clients.

**In Document:**
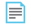
 43 CN_GD_FU_2009_T

P2 : pour moi c’est facile à réaliser, parce que le premier souci d’un homme, c’est la santé. Ce que tu manges, tu dois en prendre soin…

**In Document:**
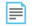
 10 CN_GD_HU_1909_T

P7 : je ne parle pas de n’importe quel animal hein, mais pour le poulet, il y en qui lorsque leur poulet est malade et tend à mourir, ils lui mettent le couteau immédiatement pour le manger. Ils ne le jettent pas hein, ils le mangent immédiatement… Ah mais oui !

Rire de quelques-uns dans la salle

P4 : si c’est un mouton, dès qu’ils constatent qu’il est malade, ils disent "tuez le en même temps on va manger rapidement" ; c’est comme ça le plus souvent.

E : autre chose ?

P2 : Mais ce n’est pas tout le monde hein…

Rire de quelques-uns dans la salle

E : ok. P7 et P4 disent que les gens tuent l’animal malade. Qui d’autre a quelque chose d’autre à dire par rapport à ce que les gens dans votre communauté font lorsqu’ils constatent que leur animal est malade ?

P2 : Ils l’envoient pour le traiter.

P7 : oui, ils peuvent l’envoyer aussi chez le vétérinaire – s’il y a les moyens d’envoyer là-bas – le visiter pour voir si la maladie dont l’animal souffre est curable ou pas. Si on ne peut pas, il pourra le savoir là-bas.

**In Document:**
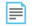
 60 CN_GD_HU1_1909_T

Que font les populations quand un animal un malade

P 1 : Ce que j’ai remarqué ici, quand c’est un animal consommable qui tombe malade, il se retrouve aussitôt dans la marmite. Si c’est animal que l’on ne consomme pas, on chasse et demande à être jeter

P8 : On demande de le préparer

P3 : La solution c’est de préparer et de manger

**In Document:**
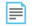
 55 CN_EN_AM_1909_T

Ça été tout à fait un problème parce que pour eux (communauté) ça c’est une source de revenu, même si tu dis le bœuf là est atteint de ça, on doit l’abattre pour n'est pas contaminer les autres. Pour eux, c’est une perte, donc on doit forcément avoir des résistances puisque la population est à majorité analphabète, eux ils ne vont pas directement voir la dangerosité de la maladie. Mais, plutôt leur économie, leur commerce d’accord ! C’est ce qui fait un peu la réticence donc là, s’il y a une maladie qui viens vraiment attaquer c’est animaux domestiques là.

**In Document:**
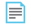
 31 CN_GD_MA2_2109_T

P4 : pourquoi c’est plus ou moins difficile, par ce que cette période l’élevage est très difficile à cause des voleurs. Si éloigne l’enclos des moutons ou chèvres, ils vont te voler la nuit. Comme il pleut actuellement, si ton enclos de chèvre est loin, même si tu as mis des tôles, ils vont les enlever et tu ne sauras rien.
