# Supplementary material for: Balancing the uncertain and unpredictable nature of possible zoonotic disease transmission with the value placed on animals: Findings from a qualitative study in Guinea
Source: PLOS Glob Public Health. 2024 Mar 28;4(3):e0001174. doi: 10.1371/journal.pgph.0001174 (PMC10977678; doi:10.1371/journal.pgph.0001174)
Supplement: Attachment — Submitted filename: PLOS One_resposes to reviewers.docx [file pgph.0001174.s004.docx]

| **Reviewer #** | **Section** | **Concerns/suggestions from reviewer** | **Response to editor** |
| --- | --- | --- | --- |
| **1** | General | A few typos: 116 Typo - executives 418 TYpo - improving | Thank you for catching these two mistakes, we have corrected them. |
| **1** | General | The first theme identifies uncertainty in animal encounters, referring to potential contact with dogs, and also contact with meat. Perhaps "encounters" referring to contact with meat products is not the correct term to use? | We rephrased the theme's title to "Recurring uncertainty and lack of control" |
| **1** | Results | In the results section, a diagram depicting how the team arrived at the two main themes showing other subthemes identified in the study would add value to understanding the background of the study | Below are the cross-cutting themes from overall study:  • Theme 1: Although people may have general basic knowledge and awareness of zoonotic diseases, they often fail to put knowledge into practice.  •Theme 2: People's traditional and religious beliefs, existing habits, and norms in their families and community may influence their ability to engage in prevention behaviors.  •Theme 3: Veterinarians play a vital role in the control and prevention of zoonotic diseases, including in the diffusion of information.  •Theme 4: Access constraints and structural barriers prevent people from engaging in healthy behaviors.  •Theme 5: People rely on multiple sources and channels of information, which can be an asset for the dissemination of information during emergencies.  •Theme 6: People live in a world with a lot of uncertainty, which can make it difficult to adopt effective prevention behaviors.  • Theme 7: People place great value on their animals for their livelihood, which influences their behaviors.  We focused on Theme 6 and 7 for this manuscript because, as noted elsewhere, they are the most novel and provide further explanation as to why people may be knowledgeable yet fail to put their knowledge into practice. To have reported on all 7 themes would have been unwieldy for a journal article. As a result, we decided to focus on the ones that were most novel and seemed to offer the greatest contribution to the literature. If you would like us to include a table to list these themes, we could do so. But to go into much more detail about the themes would not be feasible. We did further clarify the process in the manuscript, at the beginning of the Results section. |
| **1** | Results/Methods | It is mentioned that observational studies were also done on 15 participants, the results of this part of the study is not discussed or mentioned in the results section. | Thank you, we have clarified that the methodology was direct observation and provided additional detail. |
| **2** | Abstract | Line 24: Is the part of sentence after the “-“ meant to summarise all the listed categories of people? In that case maybe better replace the “-“ with “i.e”? | Thank you for this suggestion, we have edited the sentence accordingly |
| **2** | Abstract | Line 35: Do you by “infected meat” mean “meat from animals know to be suffering from an infectious disease at the time of slaughter”? “To me “infected meat” sounds like meat that has been purposively infected (spiked) with something. Please rephrase accordingly. | Thank you for catching this, we have updated all instances of "infected meat" to "unsafe meat" or "meat that is not safe" throughout the manuscript |
| **2** | Abstract | Line 29: What do you mean by “novel” in this regard? Please clarify. | We updated the word to "new" to make the sentence clearer. |
| **2** | Abstract | Line 37: I think “insights (or results) from the study” reads better. Please consider rephrasing. | Thank you for this suggestion, we have updated the sentence accordingly |
| **2** | Abstract | Line 22: There seem to be “a bridge” missing between the previous sentence mentioning preventive behaviour and this mentioning risk communication, please clarify the link between these two items. | We have added a few words to the beginning of the sentence to better connect the two ideas. |
| **2** | Abstract | Line 25: According to the material and methods it seemed you first did deductive coding and after let initial themes emerge. Is the latter what you here refer to as inductive coding? Please clarify (put the different coding steps in chronological order) and use the same terms in the different sections | We used a combined process starting with deductive coding and then employing inductive coding during the participatory data analysis process. We clarified in both the Abstract and the Methods section. |
| **2** | Abstract | Line 18-19: Please clarify how/why zoonotic diseases have been at the forefront in Guinea (due to recent outbreaks, or public health priorities or other?) | We updated the abstract accordingly, adding "due to recent well publicized outbreaks and resulting government priorities". |
| **2** | Abstract | Line 19-20: This is valid for non-zoonotic diseases as well. Please adjust the sentence accordingly. | We revised the abstract accordingly. |
| **2** | Abstract | Line 35: Priority for whom? Please clarify. | We revised the sentence accordingly. |
| **2** | General/Intro | This is a very well written paper, concerning a subject which is receiving more and more research attention and that is important for improving public and animal health and reducing antimicrobial resistance, and for achieving the related sustainable development goals. In order to warrant publication, however, some major adjustments are needed: A section describing the existing knowledge concerning what factors shape preventive behaviour for infectious diseases, especially in resource strained communities is missing from the introduction. | We have expanded on the literature concerning factors that influence behaviors related to zoonotic diseases in the introduction section. |
| **2** | Intro | Line 79-81: Yes, but a lot has been studied on these exact subjects regarding the EVD outbreak in Sierra Leone (see for example Hewlett and Hewlett 2007, Abramowitz et al 2015, Roca et al 2015). Even if the local situation in Guinea is different enough to justify specific studies on this context (and the studies on EVD don’t include the human-animal interactions), I would recommend including a short section about the insights from the numerous studies on the EVD-outbreak in Wes Africa. | Thank you, we have expanded a little bit on behaviors and behavioral drivers in the introduction section as suggested. |
| **2** | Intro | Line 86: See previous comment about LP/HPAI. | We have clarified HPAI. |
| **2** | Intro | Line 84-85: Please clarify what you mean by “priority” (for whom and why and with what purpose) and describe how the list of priory diseases was arrived at (in more details than “in collaboration” and not just by giving the reference to the report). | We have added detail on the prioritization process. |
| **2** | Intro | Line 86: I am confused what is meant by human anthrax in this regard? Please clarify, including if all prioritized disease were only prioritised by the public health authorities for disease manifestations in humans? | The prioritized diseases under GHSA (by definition as zoonoses) can exist in both humans and animals and spread between them, but countries prioritize based on their impact on human health as well as economic or environmental impacts, thus taking into account animal health. As such, we have removed the word "human" in the sentence to avoid confusion. |
| **2** | Intro | Line 82: What kind of “dearth” do you refer to here? The same kind as in the previous sentence (human-animal inter actions, societal and economic drivers of beahaviour etc) or do you refer to lack of epidemiological data (prevalence, incidence etc)? Please clarify. | We have updated this sentence for clairty by adding the following "preventative behaviors and". |
| **2** | Intro | Line 62-63: Do you refer to both high and low pathogenic avian influensa here? Otherwise you can use the abbreviation HPAI that you previously introduced. | We refer to HPAI. |
| **2** | Intro | Line 54-57 and all through: Do you refer to the disease in humans or animals here? I think the correct term for the disease and the pandemic in humans is Covid-19, whereas the disease-causing agent is of course SARS CoV-2, which is also the name of the disease in animals (infection with SARS CoV-2). Please clarify. | Thank you for nothing this, we agree and have updated all instances of of SARS CoV-2 to Covid-19 when discussing th human pandemic. |
| **2** | Intro | Line 57: I don’t see the need for the need for the sub-sentence “in consequential”. Please consider rephrasing. | Thank you for this comment, we have updated the sentence to improve readability. |
| **2** | Intro | Line 70-73: Please incorporate these lines together with the mentioning of the EVD outbreak in Guinea earlier in the introduction in line 60-63. | Thank you for this comment, we moved the sentence at line 63 down to line 70. |
| **2** | Intro | Line 85: Please write out abbreviations first time used. | Thank you, we have updated this. |
| **2** | Intro | Line 90-91: I presume this is a second aim/objective of the study, can you please reformulate accordingly. | It is part of the same overall aim of exploring the factors that influence zoonotic disease prevention behaviors. We reframed the sentences accordingly. |
| **2** | Intro | Line 91: I am confused to the last part of this sentence (“beyond EVD”), from the introduction there is nothing indicating that study only focuses on EVD? Please clarify. | We removed "beyond EVD". That phrase was initially there because there is some literature on EVD but less so on other PZDs. |
| **2** | Intro | Line 65-71: I think the jump in discourse from the role and responsibility of organization (international, national, regional, community based) in disease preventions to that of individuals is very important for the objectives of the study and deserves to be better described. Please develop this section a bit more. | Thank you for this comment. We have expanded the section more. |
| **2** | Methods | Line 106: Such interviews with individuals purposefully selected are commonly called key-informant interviews. Were these individuals key informants | While these individuals were selected based on their expertise with respect to the research questions and their communities, we asked them for their personal opinions and experiences, not only to represent the communities on which they were experts. Thus, we preferred the term in-depth interview. |
| **2** | Methods | Line 140-143: Please rephrase these sentences, this step seems to be an important part of the data analysis, but now it seems to be described partly as a training? | We have rephrased and added detail and references about the workshop, which is not a training but a collaborative analysis exercise. |
| **2** | Methods | Important information is further missing from the materials and methods-section, making it impossible to verify if the conclusions drawn from the study are valid: the sample selection process need to be described in much more detail as need the interview situation and methodology used during the interviews and focus group discussions, including if any interview protocols were used and how group dynamics and power balance were handed in the focus groups, and how the focus groups were composed (homogenous/heterogenous and considering which factors). If interview guide were used these needs to be annexed. | We have added detail in the methods section related to the sample selection process, interview protocols, topics covered in the guide, and training. |
| **2** | Methods | Line 138: Please provide a more detailed (manufacturer, country) reference for the software used. | Atlas.ti software is produced by the ATLAS.ti GmbH company headquartered in Berlin, Germany. |
| **2** | Methods | Line 138: Does this mean that all transcripts (of individual interviews and focus groups) were imported to the software? Please clarify. | Clarified that all transcripts were imported to the software for organization. |
| **2** | Methods | Line 140: Is the nationality of the senior research relevant for this section? | We removed the nationality. |
| **2** | Methods | Line 144: Was this all transcripts coded according to the initial coding framework mentioned on line 136, or parts of the transcript that fitted the initial coding? Please clarify. | Participants received coded output from the transcripts, not the entirety of the transcripts. This has been clarified in the manuscript. |
| **2** | Methods | Line 146: Are the “key themes” the same as the “novel themes” discussed in the results section? | We have added that we selected two themes from among the seven initial cross-cutting themes. |
| **2** | Methods | Line 103: Please describe the selection process in much more detail, did you for example select study sites within the three selected regions? How? How was the stratification urban-rural mentioned below done? | We have added detail on the selection process. |
| **2** | Methods | Line 109: I have never seen the expression “animal handlers” in these contexts before (as far as I know the expression is most commonly used for example for someone handling animals at a zoo or a circus). Maybe better use “animal owners or care-takers”? And for the butchers, was that the only category of stakeholders in the livestock value chain apart from farmers that were included? Why? Please clarify the selection criteria and process. | The word we used in French is "manutentionnaire", which does translate to handler and has the connotation of working with animals professionally (as opposed to an animal owner). We feel comfortable keeping the term but have added an explanation of the role and that it includes hunters and sellers as well. |
| **2** | Methods | Line 135: Please include some references to the methodology used for the analysis. | We have added references to the analysis methodology. |
| **2** | Methods | Line 108: What categories of veterinarians were these? If they were animal health providers, were they all graduated veterinarians (farmers sometimes refer to all animal health providers including community-based animal health workers as “veterinarians”). Please clarify. | We have added the term "formally trained" to clarify that these were formally trained animal health providers. |
| **2** | Methods | Line 118: Please clarify how individual participants were invited and by whom. | We have added detail in the "study sample" section on the recruitment process. |
| **2** | Methods | Line 108: Please clarify the inclusion/exclusion criteria for “the general public”. Did they need to own/take care of animals/have animal contact for example? | We have added inclusion and exclusion criteria in lines X. Participants from the general public need only be aged 18 and over. Individuals living anywhere in Guinea likely have some exposure to domestic, farm, or wild animals but we did not use this as a screening question, as we were simply interested in the perspective of "everyday Guineans". |
| **2** | Methods | Line 122-123: Did interviews and focus groups follow a topic guide or interview protocol? What was the topic areas covered? What was observed during the observations? Please clarify. | Interviews and focus groups were conducted using a semi-structured guide. We have added in information on the topics covered during data collection. |
| **2** | Methods | Line 98: Should this be in “the three respective regions” or do you presume that the results can be generalised to country level? | Thank you, we have added that clause. |
| **2** | Methods | Line 107: As “veterinarians” are mentioned on the next line, I presume these are public health providers? | We have reworded the sentence to include veterinarians with other health providers. |
| **2** | Methods | Line 95: Please describe how and why these three regions were selected. | We have added detail on the site selection. |
| **2** | Methods | Lime 92: I didnt look into the author instructions regarding subsections, but most commonly this section is referred to as “Materials and methods”. Please check this up and change if called for. | The author instructions indicate that the sub-sections can be renamed and presented in any order, so we prefer to keep this section as "Methods" |
| **2** | Methods | As the article is written in English and quotes are in English, and interviews were translated and transcribed in French, it needs to be stated at what point in the analysis the quotes were translates, and if the translation is verbatim from the transcripts or not. | We have added that the translations were done in the process of writing the report |
| **2** | Methods | In the first part of material and methods 3 activities are mentioned; individual interviews, focus group discussions and participant observations. A more detailed description of the methodology (including inclusion/exclusion criteria for sample selection and if any observation protocol was used etc) | We have added inclusion and exclusion criteria in lines and more detail about the process of direct observation. |
| **2** | Methods | Line 113: As it is mentioned that the research group consisted of both males and females, were gender aspect considered in any specific way during the data collection? Please clarify. | We have clarified that while we attempted to match the gender of the interview to the participant(s), the logistics made it complicated and we found it acceptable to mix genders considering the questions were not sensitive (for example, not about sexual or reproductive health topics). |
| **2** | Methods | Line 138-140: If the members of the research team mentioned are among the authors of the paper, please include the initials or author-list order instead. | We made this change. |
| **2** | Methods/Intro | Further needed is a description of the three study areas and the people living there including the local context of public and animal health status and prevention, animal husbandry, and not the least, the poverty situation! It is actually never mentioned in the introduction that Guinee is a low-income country, and how this affects both the resources allocated to public and animal health authorities for preventive work, peoples’ dependance of livestock for their livelihoods, or the actions people are able to take in connection to infectious diseases. This needs to be emphasized. | We have modified the methods to describe the study areas. |
| **2** | Results | Line 295: Please see previous comment regarding using the term “infected meat”. | We made this change. |
| **2** | Results | Line 286: Some parts of the text which seems not to be a quote are in italics, please check this. | Thank you for catching this, we have updated the formatting accordingly. |
| **2** | Results | Line 155: Please remove “interesting” from the results section, if the results are interesting or not can be mentioned in the discussion. | We have removed "interesting" from the results section. |
| **2** | Results | Line 163: An “and” seems to be missing. Should it be “sufficient for”? | We have removed "unsurprisingly" from the results section. |
| **2** | Results | Lnie 201: Please remove “unsurprisingly” from the results and return to such judgments in the discussion, if necessary. | We have removed "unsurprisingly" from the results section. |
| **2** | Results | Line 217-220: Please put the quotation in italics. | We have put all quotations in italics, thank you for catching this oversight. |
| **2** | Results | Line 268: In the title this theme is called “Importance of animals for”, which I believe is correct. Please align throughout the manuscript. | We have updated the title. |
| **2** | Results | Line 203: By “health” of the meat, do you mean “safety” (as in food safety), or the health of the animal from which the meat derives? Please clarify. | We changed "health" to "safety". |
| **2** | Results | Line 331 (and other places): Along the same lines as for “infected meat”, do you mean “meat from healthy animals” or meat that is specifically healthy/beneficial for the health? Please clarify. | We changed the wording to "meat that is safe for consumption". |
| **2** | Results | Line 332: For some quotes you indicate the gender of the respondent, for some not, please be consistent in this matter. | We only added gender when it was from a focus group discussion of the general lay public. For those focus groups, we had male-only discussions and female-only discussions. We thought it was important to specify, for these groups,whether they came from the male or female discussions. |
| **2** | Results | Line 202: Half of the focus groups or half of the participants in a specific focus group? If the fist, please describe in material and methods how consensus was reached. | It was half of the focus groups. We corrected the sentence and expanded the methods to further clarify. |
| **2** | Results | Line 313: Please change to “a focus group”. | I'm not sure what you are referring to. My Line 313 does say "focus group". |
| **2** | Results | Table 1: Please clarify (see previous comment) for both the human health and animal providers if they were professionals or community-based health workers. Please specify what is a vendor (of meat or live animals?). Why are hunters and transporters grouped together? See previous comment about “the general population”. What is a “Media professional”? Please provide the gender distribution also for the other respondents apart from the “general population”. | Human health and animal health providers were formal health professionals. A vendor could be of live or dead animals, but were distinct from a butcher in a market. Hunters/transporters were a smaller group of individuals. A media professional was someone that works in the media. We only had general population gender distribution because that was how we sampled for the focus group discussions. For the other categories, the gender was overwhelmingly male, but we did not classify according to gender. |
| **2** | Results | I would further recommend to reduce the number of quotes in the results section, to chose a couple for each novel theme. | We thank you for this suggestion, and understand that there are a large number of quotes in the results section. However, after review, we were not able to identify any quote to get rid of since each is rich and provide an essential piece to illustrating the data. |
| **2** | Results | Line 149: Please mention how the participants/activities were divided across the three regions as well as min-max participants/focus group. | The participants were largely equally divided across the three regions. There were 8 in-depth interviews per region and 5 participant observations concudcted per region. Of the 8 focus groups conducted per region, the total number of participants ranged from 7-10 participants per group. The total number of focus group participants was 61 in Conakry, 75 in Kankan, and 69 in N'Zerekore. This information has been incorporated in the Results. |
| **2** | Results | Line 154: Please verify the sentence for grammar and synthax. I presume these results are from the participant observations, but that is not clear, please clarify this. | The sentence was revised. |
| **2** | Results | Line 155: Are the “novel themes” the same as “key themes” mentioned in the previous section? Information/result about the initial coding framework/codes used, initial themes (are these the same as what is commonly called emerging themes?), and the key themes emerging after the discussions in the plenary discussion of the participatory data analysis workshop is missing. Please add. | We have modified the manuscript to clarify and distinguish between the overall cross-cutting themes and the two key themes of interest for the current manuscript. |
| **2** | Results | Line 155-156: This seems to belong to materials and methods/data analysis? Although I don’t understand where in the described data analysis process it was performed. Please clarify. | The text was revised to further clarify. Because it was not part of the methods outlined in the Methods section, the notion of having focused on two of the themes for this manuscript remains as part of the introduction to the Results section. |
| **2** | Results | Line 159: I would suggest using the same wording for the novel themes in the result section and in the title. Please consider rephrasing in one of the places. | We have modified the manuscript to clarify and distinguish between the overall cross-cutting themes and the two key themes of interest for the current manuscript. |
| **2** | Results | Line 160-164: Is this a summary of this theme? If not, some information seems to be repeated on line 165 and onwards, please check this. | Yes. These lines introduce the theme through a summary of sorts. The following paragraphs provide a more detailed explanation of the theme. |
| **2** | Results | Line 165 and 269; Were the data analysed according to the different regions? If so please describe this in the M&M-section. | The final data analysis culminated in the cross-cutting findings which were across the different regions. |
| **2** | Results | Line 177: The first part of this quote seems not to be related to uncertainty but to stakeholder cooperation? Please clarify. | We removed the first part of the quote and only left the portion specific to the uncertainty. |
| **2** | Results | Line 201: Did the participants really advocate “slaughtering”, or rather euthanizing or killing/putting down? Please clarify. | We changed "slaughtering" to "euthanizing". |
| **2** | Results | Line 254: “Health agent” has not been mentioned as a category of participants before. Please clarify. | We changed "health agent" to "human health provider". |
| **2** | Results | Line 312: This sentence reads like any kind of bushmeat poses the same threat concerning zoonotic diseases as meat from sick livestock, which I find controversial. Please clarify. | We revised this sentence, removing the mention of bushmeat. The idea is that individuals may consume meat that they either know is sick or that they are not sure where the animal came from or whether the animal is safe for consumption. |
| **2** | Results/Discussion | Both the results section (if the quotes are not included) and especially the discussion are short and need to be developed. | The quotes are key exemplars of the data that comprise the results. Much like providing odds ratios and p-values, quotations are essential to bring the results to life for the reader. The length of the results is appropriate given the journal and similar other qualitative manuscripts. |
| **2** | Results/Methods | Another shortcoming is that the results from some of the analytical steps described in the material and methods are not present in the results section making it difficult to understand how the “novel themes” emerged. | We have added detail to the description of the analysis workshop. Due to space limitations, we are unable to document in a detailed way the evolution of each theme throughout the framework analysis process, however we have added detail on how we selected the two novel themes from among the themes identified in lines XX. |
| **2** | Results/Methods | the results from the participant observations are however missing from the manuscript | The participant observation data was included in the review of notes and transcripts. Therefore, the data analysis process incorporated the data from these activities as well. |
| **2** | Title | Please consider rephrasing the title, I have re-read it many times but still can not quite understand it. Maybe just remove “as” and “on”? And why “recurring”? | We have revised the title. |
| **2** | Discussion | Line 400: Please describe the intended use of the focus group guides (and that you had any! And annex them) in the materials and methods section, and describe how you dealt with the problem you describe here. Otherwise the validity of the data can not be assessed. | We describe the use of guides in the Methods section. The discussion of the minor problems we faced is appropriate in the limitations. |
| **2** | Discussion | Line 401-404: It seems focus groups included participants being asked about certain behaviours. This is also new, and very interesting information that needs to be given in the materials and methods section. | We have added a table in the data collection section to list the specific behaviors explored. |
| **2** | Discussion | Line 398: Which study component? Please clarify. | We clarified that the study component in question was the use of questions about perception of what other did rather than asking about the respondents’ own practices. |
| **2** | Discussion | Line 379: I think the sentence would read nice if you replaced “outside Guinea” with for example “in other contexts (or settings or countries). | Thank you for this comment, we have replaced "outside Guinea" with "in other contexts". |
| **2** | Discussion | Line 416: “Of the effectiveness” seems redundant in this sentence. I am also not entirely convinced that the data supports this statement. Please clarify. | We updated the sentence. |
| **2** | Discussion | Line 421: What do you mean by “multiple related”? Do you refer to that hygiene or other preventive measures usually protect against several infectious diseases? Please clarify. | Thank you for this comment, we removed "multiple" from the comment to make it clearer. |
| **2** | Discussion | Line 423-427: This is already said on line 357-358. Again also consider how the results are representative. | Thank you for this comment, we removed the entire sentence given its repetativeness |
| **2** | Discussion | Line 425: What do you mean by “helpful”? Not the same as in the sentence on line 423-425? Please clarify. | We changed the word "helpful" to "inform" to be more specific. Additionally, in response to your previous comments, we made the statement more general to avoid making conclusions that are general to all of Guinea, "In addition, study findings can inform future poligies programs, whether for the current or future priority zoonotic diseases, both in similar populations Guinea and other countires in West Africa." |
| **2** | Discussion | Line 357: What do you mean by “shed light on possible implications”? Please clarify this sentence. | Thank you for this comment, we have combined the two senteces of this paragraph to be more specific:   "The cross-cutting themes in this study have helpful implications to inform and improve current and future programs aimed at the prevention, control, and surveillance of both current priority diseases of interest for Guinea also future potential zoonotic diseases outbreaks and emerging zoonoses." |
| **2** | Discussion | Line 358-360. What do you mean by “helpful for the priority diseases”? Helpful in what way? For improving control? Please clarify. | See above comment |
| **2** | Discussion | Line 360: What do you mean by “future zoonotic diseases”? Emerging zoonoses? Or future outbreak of zoonotic diseases? Please clarify. | See above comment |
| **2** | Discussion | Line 376: Please remove an abundant “information of”. | Thank you for cathing this, we have removed "information of" from the sentence |
| **2** | Discussion | Line 379 and 422: Given the restricted geographical coverage and skewed gender balance in the sampled population, please consider if your results are representative for all Guinean families/in Guinea. | Thank you for this comment, we have attempted to soften the language, so it does not appear to be representative of all of Guinea. For the first instance, we added, "some people," and for the second instance we added "among similar populations". |
| **2** | Discussion | Line 401: What do you mean by “intended audience”? Focus group participants? Please clarify. | We changed the first instance of "audience" to "participants" to be clearer about who we are refering to in this sentence. |
| **2** | Discussion | Line 387: “Own” is not needed in this construction. | Thank you for this comment, we have removed "own" from the sentence. |
| **2** | Discussion | Line 388: Please remove “likewise” and reformulate the sentence. | Thank you for this comment, we have removed "likewise" from the sentence. |
| **2** | Discussion | Line 395: I agree, please see my comment regarding the claims made on line 379 and 422 in this regard. | We added "many" to the sentence to not make a conclusion about the entire country. |
| **2** | Discussion | Line 410: What do you mean by “ecosystem” here? I can not see that the paper is about ecosystems? | We agree that the wording was confusing and added updated the sentence to read, "Two key recommendations came from the findings of this study. First, policies and programs should work to reduce uncertainty and make it easier for people to safeguard their investment and livelihood found in animals". |
| **2** | Discussion | Line 410: Is it really possible to remove uncertainties in the livelihoods for most (poor) Guineans? I think “reduce” would be more fitting in this regard. | See above comment. |
| **2** | Discussion | Line 414; I believe that communication is always multi-(or at least bi-) directional, otherwise it is simply information. Please consider rephrasing. | Thank you for this comment, we removed "multi-directional" from the sentence. |
| **2** | Discussion | Line 415. Two “support” on the same line, please rephrase the sentence. | We changed the sentence. |
| **2** | Discussion | I think the policy recommendation could be better expressed in for example a policy brief or similar than in a scientific paper, and suggest removing at least part of that section, leaving the conclusions of the study. | Thank you for your suggestion. We believe that both policy and programmatic implications are relevant for the readership of the journal. As a result, we have decided to leave the recommendations as is. |
| **2** | Discussion | Line 358: Are “the cross-cutting themes” the same as “the novel themes” mentioned in the results, or as the “key themes” mentioned in materials and methods, or something else (what in that case?)? Please clarify. | The two themes presented in this manuscript were from a larger set of cross-cutting themes that emerged from the participatory data analysis workshop. Two themes helped to uncover reasons behind a gap between knowledge about zoonotic diseases and engaging in preventive behaviors. The authors felt that these two key themes were the most novel and of greatest contribution to the field. Therefore, we decided to focus the manuscript on these two themes. We have updated the manuscript to clarify. |
| **2** | Discussion | Line 365-367: Please don’t repeat results in the discussion. | We brought up results in the discussion to ground them in the larger body of literature and knowledge. We feel it is, therefore, necessary to summarize the results in order to contextualize the findings. |
| **2** | Discussion | Line 397-399: This are very interesting methodological aspects, please include them in the material and methods. I also get a bit confused as you in the result section several times refer to differences in expressed knowledge of preventive actions and behaviour. Please clarify in both sections. | Added to methods, " Focus group discussion guides framed questions around what participants perceived that other members of their community would do or believe, rather than what the participants themselves would do or believe." |
| **2** | Discussion | Line 420: See previous comment about “priority zoonosises”. | We modified the sentence. |
| **2** | Discussion | Line 423: Of interest for whom? Please clarify. | We modified the sentence. |
| **2** | Discussion | Line 408: I am a bit hesitant to include (policy) recommendation in a research paper. I would find that more fitting in a separate policy brief (based on the research at hand). Please consider changing this section. | We understand your hesitation to include policy recommendations in papers. Our recommendations, however, combine both policy and programs and are appropriate for the readership of this journal. We believe that one of the most important things that research can do is to better inform future policies and programs, and therefore it is relevant to include them in the paper. We have modified the recommendations slightly to better reflect our perspective. |
| **2** | Discussion | Line 361-363: What about the data from the participant observations? Please clarify. | The participant observation data was included in the review of notes and transcripts. Therefore, the data analysis process incorporated the data from these activities as well. |
| **2** | Discussion | Line 379-383 and in other places: Please consider the use of the word “behaviour”. In some instances I think you rather refer to for example “preventive actions”. Especially constructions such as “to behave” can easily read as the researcher taking a top-down position. | As social and behavior change experts, we feel that it is appropriate to talk in terms of behavior. Our behaviors of interest for this manuscript are actions that people can take to prevent disease. It is appropriate to talk of these actions as behaviors. There was one place in the manuscript where we use the term "to behave" and we have removed that from the manuscript. |
