# Supplementary material for: Balancing the uncertain and unpredictable nature of possible zoonotic disease transmission with the value placed on animals: Findings from a qualitative study in Guinea
Source: PLOS Glob Public Health. 2024 Mar 28;4(3):e0001174. doi: 10.1371/journal.pgph.0001174 (PMC10977678; doi:10.1371/journal.pgph.0001174)
Supplement: Attachment — Submitted filename: Responses to reviewer comments_4-23.docx [file pgph.0001174.s005.docx]

| **Responses to reviewer comments** | | | |
| --- | --- | --- | --- |
| **Comment #** | **Reviewer #** | **Concerns/suggestions from reviewer** | **Response** |
| 1 | 2 | As I pointed out in the first review, the result section is over-crowded with quotes. You need to remove at least half of these (I have pointed out some examples of quotes illustrating similar themes, but not for all instances). Instead of (almost) only displaying the results as quotes you need to develop your themes (main themes and cross-cutting themes) and the analysis according to the framework you describe. | The manuscript comes from a qualitative research study, which aims to synthesize and present the opinions and experiences of over 200 individuals and over 100 hours of conversations/observations. Participants' words represent the data that we collected. After completing our analysis and coming up with the themes, the quotes from participants justify our interpretation of the data. We feel strongly that including the quotes from participants in the results grounds the manuscript in the data itself and not just in our interpretation. We included quotes that are necessary to support our interpretation. Much like in a quantitative manuscript you would use statistical output to justify your interpretation of the data to the reader, the quotations we selected from the countless pages of transcripts are what are necessary to justify our interpretation of the qualitative data to the reader of our manuscript. |
| 2 | 2 | A bit in a similar way (and as I also pointed out in the first review) the discussion sections is to short and to some extent shallow, and needs to be developed, in particular referring to how you discuss your results against the existing literature (instead of repeating results). A big part of the already short discussion is further policy recommendation (starting already at the end of section just before “limitations”). You further need to discuss also the limitations of the study against the existing literature. Regarding the issue of including policy recommendation in a scientific article, I support the argument that one of the most important tasks of research is to contribute to policy change, I however insist that this is better done is separate fora, each adopted to the intended purpose. You can rewrite the recommendation section and include much of the same information, but in the form of a discussion, appropriate for a scientific journal. | We have expanded the discussion section and have incorporated additional framing in reference to other research literature. |
| 3 | 2 | The revised title reeds better but is still quite a mouth-full, especially having part about “uncertainties” and “importance” in the same sentence. I would recommend another try at finding a more fitting title. | We worked on making the title more succinct yet descriptive of the manuscript's focus. The revised title now reads "Balancing the uncertain and unpredictable nature of possible zoonotic disease transmission with the value placed on animals: Findings from a qualitative study in Guinea ". |
| 4 | 2 | Line 18: you mentioned in a response to one of my queries on the first version of this manuscript that you were referring to highly pathogenic avian influenza. Please change all through the manuscript. | We have added "highly pathogenic" in the abstract and all other mentions are HPAI, except references. |
| 5 | 2 | In the abstract, we removed the term and replaced it with two examples of the types of individuals that we later refer to as an animal handler. We added the French term in brackets the first time it is used in the main manuscript. From there on, we continued to use the term "animal handler". The term “people taking care of/looking after animal/interacting with animals” is too general and would basically include all of the types of participants in our study. The purpose of the study was not to make precise differentiations or comparisons among different types of individuals involved in the raising or selling of animals. | In the abstract, we removed the term and replaced it with two examples of the types of individuals that we later refer to as an animal handler. We added the French term in brackets the first time it is used in the main manuscript. From there on, we continued to use the term "animal handler". The term “people taking care of/looking after animal/interacting with animals” is too general and would basically include all the types of participants in our study. Apart from the changes described, we kept the rest of the mentions in the manuscript as "animal handlers" because the purpose of the study was not to make precise differentiations among the specific types of individuals that deal with the raising/selling of animal products for financial gain. |
| 6 | 2 | Line 29: From your additional description of the methodology, this doesn’t seem to be a correct description? Didn’t 7 themes arose, butarise, and you choose to explore 2 (the most novel)? | We have updated the abstract to make it more explicit that we are focusing on the two most novel themes out of the set of key themes that came up during the analysis |
| 7 | 2 | Line 33-35: Here you mention “vaccinating animals” and “selling unsafe meat” as example of preventive behaviors. In order for that to make sense you need to explain your definition of “prevention”, especially in connection to infectious diseases, or choose another wording (referreferring to the term and definition of “preventive medicine”). The first is of course a classic preventive measure, protecting herds against infectious diseases. Do you mean here that “selling unsafe meat” is a preventing behavior as it protects the farmer from a possible financial loss if not selling meat from infected animals? That is of course true, bit grouped together with a traditional infectious disease preventive measure like that it needs explanation/rephrasing. | Thank you for identifying this inconsistency. We have reworded, giving the example of "avoiding the sale of unsafe meat" is the behavior that would prevent disease. |
| 8 | 2 | Line 47: “2,5 billion illnesses” is difficult to grasp, it is more useful to measure the impact in DALYS. I believe some more recent references with impact might be available. | We went back and reviewed the literature again to see what we could find. Recent research on DALYs (and zDALYs that consider the economic impact of lost animal production) has mostly been disease specific. We found countless examples of recent peer-reviewed journal articles which use estimates like what we have used to demonstrate the burden of zoonotic disease. We have kept the current wording but did incorporate an estimate of the global burden of rabies, including DALYs, to provide a specific example. |
| 9 | 2 | Line 48-59: Please check how you capitalize disease names, and be consistent through the manuscript. Given as this is not a popular scientific paper I would recommend removing “bird flu” and “mad cow disease”. | Thank you for pointing this out. We have edited to follow the guidelines that each letter in acronyms should be capitalized whereas disease names and viruses should be lower case unless they contain a proper noun. |
| 10 | 2 | Line 51: There seems to be either a “(“ missing or a ”)” to many. | Revised, per the suggestion. Thank you. |
| 11 | 2 | Line 77: Do you mean “at” multiple levels? | Changed to "at multiple levels" |
| 12 | 2 | Line 84: I think a more frequently used term is “hens, chickensDo you mean “poultry” or maybe “poultry and other captive birds”? | We have replaced "fowl" with "poultry and other captive birds", as suggested. |
| 13 | 2 | Line 89: Do you mean “modern slaughter houses” or maybe “butcheries”? | Thank you for the comment; we have replaced the term with "slaughterhouses." |
| 14 | 2 | Line 94-95: Please rephrase this sentence. | We have rephrased the sentence to read as follows, "Meanwhile, N’Zérékoré is a densely populated prefecture in the forested region of Guinea, hunting and consumption of bushmeat common throughout." |
| 15 | 2 | Line 96: Do you mean “are” prevalent? | Revised, per the suggestion. Thank you. |
| 15 | 2 | Line 111-115: The sentence seems to be missing an item – “points to xx and yy” for what or as examples of? | Rephrased the sentence. |
| 16 | 2 | Line 125: I would recommend to remove “priority” from this sentence, it doesn’t add any extra information (as the diseases are already listed), but on the contrary adds confusion as to for whom they are prioritized. | Removed "priority". |
| 17 | 2 | Line 126-128: Please reformulate this sentence, for example starting with “and additional aims was to…” | Reformulated the sentence which now reads as follows, "An added benefit of this exploration of factors that influence zoonotic disease prevention behaviors is to help fill the above-stated gap in the literature." |
| 18 | 2 | Line 134-136: Please provide a reference for the assessment of elevated risks. | We selected the regions in communication with the National Health Security Agency (Agence Nationale de Sécurité Sanitaire or ANSS). In our conversations with them, they verbally told us which regions they wanted us to prioritize, along with the general background information about the sites mentioned in this paragraph. There is not a specific published reference to site. |
| 19 | 2 | Line 143: What do mean by “pile-sorting”? please explain and/or give a reference. | We have explained the pile sorting process in more detail in the manuscript. |
| 20 | 2 | Line 144: I think “feasibility” is enough for defining the first dimension (self-efficacy doesn’t explain anything further for me). Pease consider removing. | Removed "self-efficacy". |
| 21 | 2 | Line 159: Please include information on selection of study sites (prefectures?) in the three study regions. Unless all prefectures were included. Maybe add addition in the section about the study areas about how many prefectures each region have. | The description of the selection of the three prefectures is in the manuscript. |
| 22 | 2 | Line 168: Do you mean in “each prefecture of the selected regions” or in “each selected prefecture”? | Prefectures (Conakry, Kankan, N'Zerékoré) are the areas/regions where the study was conducted. We have replaced regions with prefectures throughout the manuscript when referring to the specific area of Guinea (i.e., Conakry, N'Zerékoré, Kankan) where we focused our study. |
| 23 | 2 | Line 169, 171 and 172: The recruitment process is not clear, “the general population” is mentioned twice, please clarify. | Thank you, we have reorganized to clarify that local neighborhood chiefs identified and recruited members of the general population, whereas leaders of livestock associations or local officials recruited the other types of participants. |
| 24 | 2 | Line 172: The age-inclusion criteria is already mentioned on line 159. Please remove in one of the places. | We have removed the age inclusion criteria in line 159. |
| 25 | 2 | Line 185-187: Please cross-check this with how the recruitment is described on line 169 and keep the information to one place. | We have consolidated the information on recruitment into the "study sample" section. |
| 26 | 2 | Line 189: Do you mean “convenient for the participants”? | We have reworded the sentence to clarify that the locations were selected to be convenient for participants. |
| 27 | 2 | Line 194: Please annex the interview and topic guides. | We have added all focus group discussion and individual interview guides as supporting information. |
| 28 | 2 | Line 221-222: I don’t think the passage about the researchers experience is necessary here. Please remove. | We have removed the description of the PI's experience in Guinea. |
| 29 | 2 | Line 224: Should one more author be indicated here as “study managers” are in plural? Better to indicate the initial first time mentioned a couple of lines earlier. | Thank you for catching the typo; we have replaced with singular study manager. |
| 30 | 2 | Line 228: What do you mean with “by region”? That the participant representing one region formed a group, or that each groups worked with data from one of the three regions, or both of these? | We have added language to explain that small groups were assigned to work with data from a particular prefecture. |
| 31 | 2 | Line 232: Do you mean “saturation was reached”? | We have revised the sentence as suggested. |
| 32 | 2 | Line 235: See previous comment about selection of study sites within the regions. Or did you choose participants from all over the regions? | We used a purposeful sampling for each prefecture, as described in the manuscript. |
| 33 | 2 | Line 236 -238: Please write numbers smaller and equal to eleven in letters. | Revised, per the suggestion. Thank you. |
| 34 | 2 | Line 240: This seems to be the end of a first section about number of participants. Please indicate the start of a new section. Somewhere around here it would be nice to list, maybe in a table, all themes (all 7), the 2 you choose to include in the article and the cross-cutting themes. | The beginning part of the results is a general overview and does not seem to have a distinct separate section. We did, however, started a new paragraph at Line 241. We also included a table with all 7 cross-cutting themes, highlighting the two novel themes which are the central focus of this manuscript. |
| 35 | 2 | Line 249: A blank line seems to be missing under the table. | Revised, per the suggestion. Thank you. |
| 36 | 2 | Line 267-269: I think this quote is representative of the four quotes on line 260-269 and 281-283, please keep only one. | Within the manuscript, we included different quotes on a similar theme to illustrate that the idea was expressed by different types of participants and in differerent prefectures. All of the quotations we selected are necessary in order to demonstrate the cross-cutting nature of the theme. |
| 37 | 2 | Line 270: A “that” seems to be missing in this sentence. | Revised, per the suggestion. Thank you. |
| 38 | 2 | Line 293-308: These 3 quotes seem to allure to the same theme, please keep only one. | Within the manuscript, we included different quotes on a similar theme to illustrate that the idea was expressed by different types of participants and in different prefectures. All the quotations we selected are necessary to demonstrate the cross-cutting nature of the theme. |
| 39 | 2 | Line 320-325: These 3 quotes seem to allure to the same theme, please keep only one. | Within the manuscript, we included different quotes on a similar theme to illustrate that the idea was expressed by different types of participants and in different prefectures. All the quotations we selected are necessary to demonstrate the cross-cutting nature of the theme. |
| 40 | 2 | Line 332-339: These 2 quotes seem to allure to the same theme, please keep only one. | Within the manuscript, we included different quotes on a similar theme to illustrate that the idea was expressed by different types of participants and in different prefectures. All the quotations we selected are necessary to demonstrate the cross-cutting nature of the theme. |
| 41 | 2 | Line 369-380: These quotes seem to allure to the same theme, please keep only one. | Within the manuscript, we included different quotes on a similar theme to illustrate that the idea was expressed by different types of participants and in different prefectures. All the quotations we selected are necessary to demonstrate the cross-cutting nature of the theme. |
| 42 | 2 | Line 360-366, 386-399, 422-424: These quotes seem to allure to the same theme, please keep only one. | Within the manuscript, we included different quotes on a similar theme to illustrate that the idea was expressed by different types of participants and in different prefectures. All the quotations we selected are necessary to demonstrate the cross-cutting nature of the theme. |
| 43 | 2 | Line 404-419: These quotes seem to allure to the same theme, please keep only one. | Within the manuscript, we included different quotes on a similar theme to illustrate that the idea was expressed by different types of participants and in different prefectures. All the quotations we selected are necessary to demonstrate the cross-cutting nature of the theme. |
| 44 | 2 | Line 432-444: These quotes seem to allure to the same theme, please keep only one. | Within the manuscript, we included different quotes on a similar theme to illustrate that the idea was expressed by different types of participants and in different prefectures. All the quotations we selected are necessary to demonstrate the cross-cutting nature of the theme. |
| 45 | 2 | 447-448: Please either remove “presented above” or replace with naming the two themes. | By combining the first two sentences of this paragraph, as requested, we eliminated this issue. |
| 46 | 2 | Line 446-450: These two sentences seems repeatative. Please rephrase, or maybe remove the first sentence. | Thank you for the comment. We have rephrased the first two sentences of the paragraph. |
| 47 | 2 | Line 451: Sufficient to say “the data” without repeating the methodology. | We removed mentioning the methodology in the sentence. |
| 48 | 2 | Line 453: Was “unpredictability of disease control” a theme/a result? Please clarify this. | Uncertainty/unpredictability is one of the two themes in the study. |
