# Supplementary material for: Balancing the uncertain and unpredictable nature of possible zoonotic disease transmission with the value placed on animals: Findings from a qualitative study in Guinea
Source: PLOS Glob Public Health. 2024 Mar 28;4(3):e0001174. doi: 10.1371/journal.pgph.0001174 (PMC10977678; doi:10.1371/journal.pgph.0001174)
Supplement: Attachment — Submitted filename: Responses to reviewer comments_8-23.docx [file pgph.0001174.s006.docx]

| **Responses to reviewer comments** | | |
| --- | --- | --- |
| **Comment #** | **Comment** | **Response** |
| 1 | The manuscript has unfortunately still not reached the quality expected for publication in PGPH, and require another major/moderate revision. Many of my previous comments have been addressed in a satisfactory way, and the manuscript has improved in clarity. Some previous comments are mentioned in the rebuttal letters as taken care of while this seems not to be reflected in the text. Some changes are reflected in the tracked version but not in the clear version. Other minor issues have arisen as the manuscript has been redrafted. Quite a few of those are of language editorial or just editorial matter and could easily have been avoided by a thorough language/editing check. | Somehow the final version of the previous round of revisions did not make it to the final submission. We apologize for any inconvenience. We have taken you feedback on the latest revision and addressed them in the correct version from last round. |
| 2 | Regarding my previous comment 1, 36-43 and 46 that the authours have chosen to ignore. I am obviously aware of the basics of qualitative research methods (otherwise I would (hopefully) not be reviewing this manuscript). I am also aware about different “schools” for how to use quotes. I am not asking for the quotes to be removed, but in the (many) instances there you have several (sometimes up to four) quotes supporting the same theme or the same statement, reduce the numbers. | We would like to clarify that we did not ignore the comments made, but rather that we have a different opinion about what is important to include to justify the points that we are making from our analysis. Our rationale for keeping the multiple quotes is to illustrate how a certain point/theme was present in multiple regions and types of individuals. Including multiple quotes makes the point of triangulation of findings across regions and types of individuals. |
| 3 | Regarding my previous comment 2: The authours has done a slight rework of the discussion, but it is still very short and as I mentioned in my previous comment, neither the limitations nor the conclusion is discussed against the current literature. As I recommended, most of the conclusions could be worked into the discussion. There are a lot of literature discussing the matters raised in first part of the conclusion (lines 499-514, barriers to implementation of prevention beahaviour, the relations between livelihood and disease prevention, communication) that could be used. | We have expanded the discussion section to make a more explicit link to other relevant literature. |
| 4 | Please make sure that figure captions are “stand alone”. | We have revised the titles of the figures in the manuscript |
| 5 | The new suggested title (“Balancing the uncertain and unpredictable nature of possible zoonotic disease transmission with the value placed on animals: Findings from a qualitative study in Guinea” as per the title page) reads much better. The change does however not seem to be reflected in the manuscript. | Somehow the final version of the previous round of revisions did not make it to the final submission, including the revised title. We apologize for any inconvenience. We have corrected the title accordingly. |
| 6 | Line 25: See my previous comment on “animal handlers” (comment 5 in your numbering of my comments on the previous version), it does not seem that the clarification you mention in your answer is reflected in the text? | The current manuscript reflects our previous clarification which is as follows: "In the abstract, we removed the term and replaced it with two examples of the types of individuals that we later refer to as an animal handler. We added the French term in brackets the first time it is used in the main manuscript. From there on, we continued to use the term "animal handler". The term “people taking care of/looking after animal/interacting with animals” is too general and would basically include all types of participants in our study. Apart from the changes described, we kept the rest of the mentions in the manuscript as "animal handlers" because the purpose of the study was not to make precise differentiations among the specific types of individuals that deal with the raising/selling of animal products for financial gain." |
| 7 | Line 33-35: “The value placed on livestock may, in turn, drive and impede prevention behaviors such as vaccinating animals or selling unsafe meat”. See my previous comment regarding “selling unsafe meat” as a prevention behaviour (comment 7 in your numbering of my comments on the previous version). | The current manuscript reflects our previous clarification which is as follows: "Thank you for identifying this inconsistency. We have reworded, giving the example of "avoiding the sale of unsafe meat" is the behavior that would prevent disease." |
| 8 | Line 59: See comment 9 in your numbering of my comments on the previous version regarding capitalization of disease names. | The current manuscript reflects our previous response regarding fixing the capitalization of names of diseases. |
| 9 | Line 66-67: The sentences seem to more factors that could prevent disease transmission (vaccination, hygiene practices, care-seeking for zoonotic diseases, or how people protect themselves) than “individual behaviors that can lead to zoonotic events or zoonotic disease transmission” Please clarify this. | Thank you, we have revised this sentence to clarify, "Moreover, a variety of individual behaviors can prevent zoonotic events or zoonotic disease transmission, such as vaccination, good hygiene practices during food preparation, care-seeking for zoonotic diseases..." |
| 10 | Line 78: The sentence would read better with “in recent years” placed at the end of the sentence. | Moved "in recent years" to the end of the sentence. |
| 11 | Line 84: See my previous comment (comment 12 in your numbering of my comments on the previous version) on the use of “fowl”. | The current manuscript reflects our changing the use of "fowl". |
| 12 | Line 86: “for transmission of zoonotic diseases” seems to be missing after “risk factor”. | Added "for transmission of zoonotic diseases" after "risk factor" |
| 13 | Line 92 and 96: I presume you mean that the mentioned diseases are prevalent in animals? Please specify. It would be good to include a reference for the claims of high prevalence of the zoonotic diseases. I am very aware of the difficulties with official statistic form these kinds of contexts, but I’m sure that your claim is based on something (unpublished data, personal communication, unofficial disease reports)! When the prevalence is unknown it is also sometimes better to use “frequently occurring” or some other wording that is less epidemiologically loaded. | We removed prevalence and replaced the wording so that it ls less epidemiologically loaded. |
| 14 | Line 107: The construction “non-bush meat” is innovative, but I still suggest for example “other meat than bushmeat”. Please consider this. | Thank you, we have revised as suggested. |
| 15 | Line 112: Please consider exchanging “cross-cutting” with “general” or “basic”. | General or basic does not capture the idea that the seven themes cut across various of the behaviors of interest. We have reworded the sentence to remove cross-cutting and to be more specific that the theme touched on multiple behaviors. We also removed "cross-cutting" throughout the manuscript. |
| 16 | Line 134-137: Please move this info to the section in the introduction which describes the respective areas. | We moved these details into the introduction, as requested. |
| 17 | Line 143: I’m still confused about the pile-sorting. Is it different from “proportional piling” (which is a common tool in participatory epidemiology)? | Yes. Pile sorting is different from proportional piling. We added additional description of the methodology in the manuscript. |
| 18 | Line 155: Please rephrase particularly the second part of the sentence to improve grammar and synthax. Please change to “working day”. | "Workday" can be found in Merriam-Webster, Collins, and Oxford dictionaries. Our use of the word is grammatically correct, and we prefer to use that word instead of "working day". We did, however, rework the sentence to improve the readability. |
| 19 | Line 170-172: Please rephrase the sentence to improve grammar and synthax. | We removed the sentence and reworked the recruitment process details. |
| 20 | Line 158 (study sample): Please see comment 21 and 22 in your numbering of my comments on the previous version. Within the 3 selected prefectures, did you not do any further section of study sites before making the purposive selection of participants (you mention local community/local neighborhoods)? I.e did your 229 participants include people from the entire prefectures or only specific sites (I don’t know what is the next lower levels of administrative units, maybe sub-prefecture, village, ward or something similar)? | We did not have specific sites. Instead, the local guides from each prefecture were the ones to recruit and help select potential participants. The local guides were identified by the national veterinary services. |
| 21 | Line 168: The previous part of this section seems to concern the different methods, and here the subject of selction/mobilizing start. It might help the reader to include a blank line here to mark that this isa new topic. | There is a hard stop (new paragraph) where the new idea of recruitment begins. Line 168 is still about the selection of the sample of the various methods. |
| 22 | Line 179: “multi-day” sounds peculiar, please consider rephrasing. | We have revised to "took place over multiple days". |
| 23 | Line 180: There seems to be an abundant “the” in the phrase. | Thank you, we have removed it. |
| 24 | Line 181: Please include a reference to the data collection tools that you have annexed in supporting information (interview, FGD and observation guides) at for example this point. | We have added the reference as suggested. |
| 25 | Line 183-84: Please rephrase the sentence to improve grammar and synthax. | We have revised the sentence for clarity and explain better the reasons for mixed gender focus groups among people who work professionally with animals. |
| 26 | Line 185-188: This sentence seems to refer to selection of the study sample? Please move to the relevant section (approx. line 168-172). | We moved these details to where we describe the study sample selection. |
| 27 | Line 189: See my previous comment (comment 26 in your numbering of my comments on the previous version), it does not seem that the clarification you mention in your answer is reflected in the text? | This change should now be reflected. |
| 28 | Line 216: I presume the “research team” are all authors? If the frame work was developed by TG and NT (and not the entire research team) it seems the sentence should start “TG and NT developed….”. | We revised the sentence as suggested. |
| 29 | Line 218: Who are these study managers? Co-authours? Please clarify. | We have clarified that the initial coding was done by 4 of the data collectors, their supervisor who was the lead consultant in Guinea, and the study manager on the study team. |
| 30 | Line 221: See my previous comment (comment 28 in your numbering of my comments on the previous version), it does not seem that the clarification you mention in your answer is reflected in the text? | This change should now be reflected. |
| 31 | Line 223: Who were the participants in this analysis that were not data-collectors and how were they selected (not mentioned in sample seletion as far as I can see). Please clarify. | We expanded the description of who were the participants. In addition to the data collectors, study managers, and government staff that was identified in the manuscript, several other participants were programmatic staff of the project that funded the research. Participants of this workshop were selected by the principal investigator in collaboration with the study managers and the staff person in charge of the zoonotic disease work of the project. |
| 32 | Line 231-232: Please rephrase the sentence to improve grammar and synthax. | We have revised the sentence. |
| 33 | Line 234-247: Please format this section correctly and according to author guidelines. | We reformatted the manuscript. |
| 34 | Line 236-38: Please see my previous comment regarding writing out numbers with letters (comment 33 in your numbering of my comments on the previous version), it does not seem that the clarification you mention in your answer is reflected in the text? | We have written out numbers under twelve. |
| 35 | Line 243: Do you mean “health prevention behaviour”? | We simplified this to "prevention behaviors" to be consistent with the rest of the text. |
| 36 | Line 246: I would suggest to start the sentence with "The authours”. | Changed to "the authors" |
| 37 | Line 293, 373: There seems to be an abundant [ ]? | Removed brackets. |
| 38 | Line 446-450: The first two sentences seem to convey very similar messages. Please consider rephrasing. | We have reworked the first two sentences as suggested. |
| 39 | Line 463: A “the” seems to be missing in front of “Covid-19”. | Thank you, we have added "the" to the sentence. |
| 40 | Line 465: Please rephrase the sentence to avoid the construction with “information of information”. | We have rephrased as suggested. |
| 41 | Line 507: There seems to be an abundant “b”. | Thank you, we have removed the typo. |
| 42 | Line 518: Please rephrase the sentence to improve grammar and synthax. | We have revised the sentence. |
