# Supplementary material for: Balancing the uncertain and unpredictable nature of possible zoonotic disease transmission with the value placed on animals: Findings from a qualitative study in Guinea
Source: PLOS Glob Public Health. 2024 Mar 28;4(3):e0001174. doi: 10.1371/journal.pgph.0001174 (PMC10977678; doi:10.1371/journal.pgph.0001174)
Supplement: Attachment — Submitted filename: Responses to reviewer comments_10-23.docx [file pgph.0001174.s007.docx]

| **Responses to reviewer comments** | | |
| --- | --- | --- |
| **Comment #** | **Comment** | **Response** |
| 1 | line 128 "diseases, the frequency" - add the space after , | Made this edit. |
| 2 | line 180 remove one "." | Made this edit. |
| 3 | line 188 "participated in a training [session?]" | The trainin was not a single session but rather a training that lasted several days. We reworded the sentence to clarify and simplify. The sentence now starts, "Data collectors participated in a multi-day training during which they ..." |
| 4 | line 197 space after "." | Made this edit. |
| 5 | line 211 place "." after ")" | The words in the parentheses are a stand-alone sentence. In this instance, therefore, the punctuation mark for the sentence is placed inside the closing parenthesis, not outside. No change has been made. |
| 6 | line437 "that is safe" | Made this edit. |
| 7 | line 552 - font? | The font in the manuscript is the same in this line compared to the others in the manuscript submitted. But in the pdf, somehow the font does look a little different. No change was made, but we will double check once the manuscript is saved in pdf. |
| 8 | line 568 double .. | Made this edit. |
| 9 | Line 52; There seems to be an abundant comma after “(HPAI)”. | Made this edit. |
| 10 | Line 180, line 568. There seems to be an abundant full stop. | Made this edit. |
| 11 | Line 197: A space seems to be missing. | Made this edit. |
| 12 | Line 86-105: I understand the lack of official data, but please try to find some references (grey literature, oral communication) for at least some of the important background statements in this section. | We have modified the narrative to remove some of the statements that we could not find confirm our references. We also added several references to some of the remaining statements. |
